# What does the mediodorsal thalamus do?

**DOI:** 10.3389/fnsys.2013.00037

**Published:** 2013-08-09

**Authors:** Anna S. Mitchell, Subhojit Chakraborty

**Affiliations:** Department of Experimental Psychology, Oxford UniversityOxford, UK

**Keywords:** prefrontal cortex, memory, executive function, macaque, rodent, animal models, learning

## Abstract

Dense amnesia can result from damage to the medial diencephalon in humans and in animals. In humans this damage is diffuse and can include the mediodorsal nuclei of the thalamus. In animal models, lesion studies have confirmed the mediodorsal thalamus (MD) has a role in memory and other cognitive tasks, although the extent of deficits is mixed. Anatomical tracing studies confirm at least three different subgroupings of the MD: medial, central, and lateral, each differentially interconnected to the prefrontal cortex (PFC). Moreover, these subgroupings of the MD also receive differing inputs from other brain structures, including the basal ganglia thus the MD subgroupings form key nodes in interconnected frontal-striatal-thalamic neural circuits, integrating critical information within the PFC. We will provide a review of data collected from non-human primates and rodents after selective brain injury to the whole of the MD as well as these subgroupings to highlight the extent of deficits in various cognitive tasks. This research highlights the neural basis of memory and cognitive deficits associated with the subgroupings of the MD and their interconnected neural networks. The evidence shows that the MD plays a critical role in many varied cognitive processes. In addition, the MD is actively processing information and integrating it across these neural circuits for successful cognition. Having established that the MD is critical for memory and cognition, further research is required to understand how the MD specifically influences these cognitive processing carried out by the brain.

## Introduction

It is now more widely recognized that the episodic memory processes disrupted in anterograde amnesia involve interactions between the medial temporal lobes and the medial diencephalon (Aggleton and Brown, 1999). However, understanding how the medial thalamus contributes to memory and other cognitive functions has been much overlooked in cognitive neuroscience and neuropsychology. This mainly stems from the theoretical notion held over the past 50 years that the medial temporal lobes act exclusively as the brain's long-term declarative memory center. Despite this prevailing account, human patients can also suffer dense amnesia following damage in the medial diencephalon (diencephalic amnesia, thalamic amnesia). Brain damage that causes memory loss and other cognitive deficits in this region occurs after traumatic head injury, stroke, hemorrhage, thiamine deficiency, or chronic alcoholism (Korsakoff's syndrome). However, this brain damage is not circumscribed in clinical patients as many of the structures of the medial diencephalon (medial thalamus, mammillary bodies, and mammillothalamic tract) suffer combined damage due to their small size, close proximity to one another and fibers of passage coursing through the region. The medial thalamic structures most frequently identified as being critical for the memory deficits are anterior (AT), mediodorsal (MD), and the intralaminar (IL)/midline thalamic nuclei. The mammillary bodies and white matter fiber tracts, particularly the internal medullary lamina and the mammillothalamic tract, are also strongly implicated in human amnesic cases and animal models. Thus, the neural basis of the memory deficits associated with the medial diencephalon continues to be debated in the literature (Harding et al., 2000; Kopelman, 2002; Van der Werf et al., 2003a; Cipolotti et al., 2008; Aggleton et al., 2011; Carlesimo et al., 2011; Pergola et al., 2012; Vann, 2013).

Animal models of diencephalic amnesia are critical in helping to determine the structures that are important for memory and other cognitive processes as well as understanding the neural circuitry of this region. The emphasis of this review is on the experiments in animal models (monkeys and rodents mainly) that assess the role of the MD in memory and other cognitive processes. The review will show how this research can extend our understanding about the functions of the MD that when damaged cause some of the symptoms of the human amnesic syndrome. There is also a section on anatomy of the MD and its interconnections with other brain structures: detailing the communication within these regions is critical for understanding their overall functioning. It is important to remember that lesion studies do not show what the area of the brain that has been lesioned does, rather they show how the rest of the brain functions and compensates after brain injury to a particular region has occurred. Furthermore, we know that a single region of the brain does not act alone. Thus, the brain structures of the medial thalamus are interconnected with other brain structures, together forming integrated neural networks of cognition. The review concludes with an overview of some of the theories of MD involvement in cognition and memory, current perspectives and possible future directions to investigate.

It is an exciting time to be studying the medial thalamus and its role in cognitive processing as the work of many is challenging the long held beliefs that the thalamus is only passively relaying information from the basal ganglia, midbrain and brainstem onto the prefrontal cortex (PFC). For example, more recent neuroanatomical and neuromodulatory studies highlight how the thalamus is providing a critical role in integrating communication between the basal ganglia, thalamus, and cortex, which is challenging many long standing theoretical ideas related to the passive role of the thalamus (Haber and McFarland, 2001; Guillery and Sherman, 2002; Sanchez-Gonzalez et al., 2005; Sherman and Guillery, 2005, 2011; Sherman, 2007; Haber and Calzavara, 2009).

In addition, with advances in neuroimaging and its analyses, and different electrophysiology techniques that can help investigate functional and anatomical connectivity, the medial thalamus and specifically the MD has now been shown to influence many cognitive processes including memory, decision-making, and executive functions with comparative data across numerous species. The MD is also a critical structure linked to many neurological disorders (e.g., stroke, dementia, schizophrenia, major depressive disorder, Parkinson's disease, and Alzheimer's disease). Clearly, further research is needed on the MD to develop greater understanding of the neural mechanisms of its functioning and how it contributes to many neurological disorders.

## Anatomy of the MD

Many of the structures in the brain can go by several names and this is the case with the MD, which is also referred to as medial dorsal thalamic nuclei, nucleus medialis dorsalis, and the dorsomedial thalamus. For the purposes of this review, the structure will be referred to as the mediodorsal thalamus (MD) and at some points will be distinguished by some of its subdivisions, that is, the magnocellular mediodorsal thalamus (MDmc) or medial MD, the parvocellular mediodorsal thalamus (MDpc) or central MD, and a lateral grouping that will include the densocellular (MDdc) and pars multiforms (MDmf) mediodorsal thalamic nuclei or lateral MD (MDl).

### Cytoarchitecture

**Figure 1:**
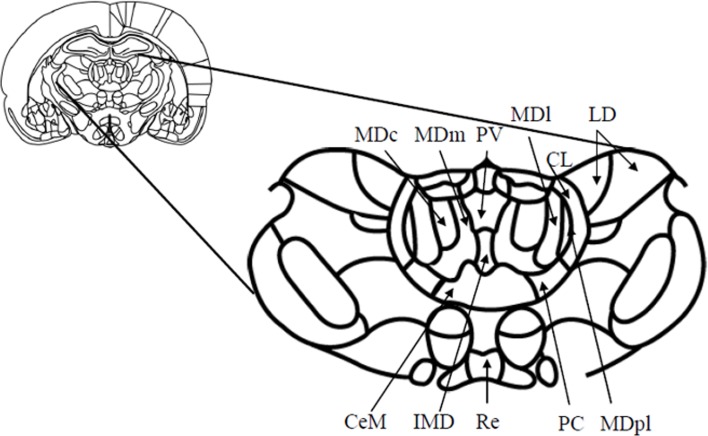
**Schematic diagram (and enlargement) of the medial aspects (Bregma—2.56 mm) of the medial thalamus in the rodent brain.** Abbreviations: CeM, center median nucleus, part of the midline nuclei; CL, centrolateral nucleus, part of the intralaminar nuclei; IMD, intermediodorsal nucleus, part of the midline nuclei; LD, laterodorsal nucleus; MDc, central subdivision of mediodorsal thalamus; MDl, lateral subdivision of mediodorsal thalamus; MDm, medial subdivision of mediodorsal thalamus; MDpl, paralamellar subdivision of the mediodorsal thalamus; PC, paracentral nucleus, part of the intralaminar nuclei; PV, paraventricular nucleus, part of the midline nuclei; Re, reuniens. Adapted from Paxinos and Watson (1998).

**Figure 2:**
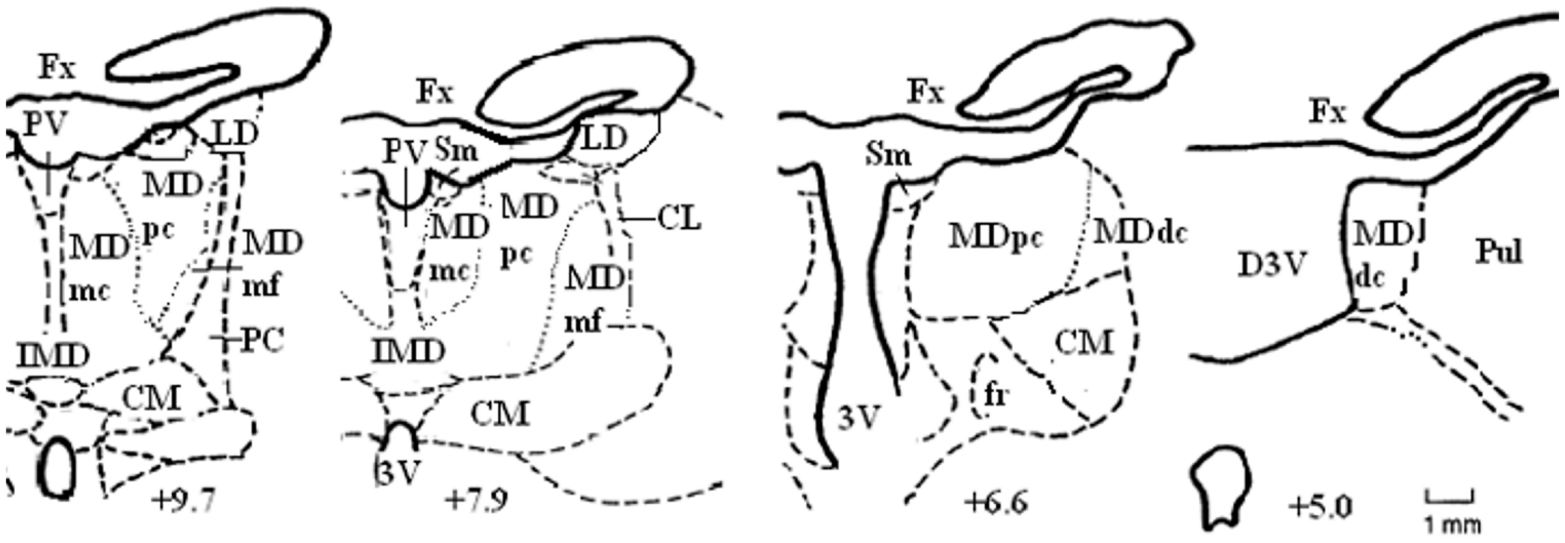
**Schematic diagrams of some of the coronal sections located approximately IA+9.7, +7.9, +6.6, and +5.0 through the rostrocaudal extent of the medial thalamus in the non-human primate brain.** Abbreviations: CM, centromedian nucleus; Fx, fornix; Pul, pulvinar; Sm, stria medullaris. Adapted from the atlas of Olszewski (1952).

The MD is considered the largest of the nuclear structures in the medial thalamus, and it is most developed in primates, especially humans. The increase in the size of the MD in phylogenetic evolution parallels that of prefrontal, association and cingulate cortices (Bentivoglio et al., 1993; Jones, 1998). In rats, the MD is relatively heterogeneous with four main subdivisions identified (see Figure 1). These are the medial, central, lateral, and paralamellar segments (Krettek and Price, 1977; Groenewegen, 1988). The boundaries of each segment are somewhat well defined, especially between the central and lateral segments. The dendrites of the cells in each of these two segments tend to be confined to their respective regions and the lateral segment stains more heavily for acetylcholinesterase (Price, 1995). In primates, the four subdivisions are more easily recognizable (see Figure 2): a magnocellular subdivision (MDmc) occupies the most medial and rostral part of the MD and is considered equivalent to the medial segment in rats. The parvocellular (MDpc) subdivision is located within the central part of MD throughout the rostrocaudal extent. The other two subdivisions, densocellular (MDdc) and pars multiforms (MDmf) are located in the lateral part of MD with the MDmf situated in the rostral part and MDdc situated in the caudal part of the MD (Jones, 1985; Bentivoglio et al., 1993; Bachevalier et al., 1997).

### Neural connections of the mediodorsal thalamus

#### Prefrontal cortex afferents and efferents

In rodents and non-human primates, there are substantial reciprocal interconnections between the PFC and the MD (Krettek and Price, 1977; Goldman-Rakic and Porrino, 1985; Groenewegen, 1988; Ray and Price, 1993; McFarland and Haber, 2002; Xiao et al., 2009). Higher order thalamic structures, like the MD (and the pulvinar) receive inputs from different cortical layers. The majority of projection neurons to the MD originate from layer VI and V (Giguere and Goldman-Rakic, 1988; Yeterian and Pandya, 1994; Xiao et al., 2009); mainly from within the deep regions of these layers. The cortical layer V pyramidal neurons also have branches of long descending axons going to motor centers (Guillery, 1995; Guillery and Sherman, 2002). Guillery also proposed that these higher order thalamic nuclei play a key role in cortico-cortical communication and higher cortical functioning (Guillery, 1995). Thalamic neurons innervated by cortical layer VI project focally to the middle cortical layers and thalamic neurons innervated by cortical layer V project widely to the superficial cortical layers which are involved in cortico-cortical communications (Jones, 1985; Xiao et al., 2009). In addition, there are nonreciprocal components to the thalamo-cortical links, indicating a dual role for the MD in integrating basal ganglia outputs within specific cortical circuits (see below) (McFarland and Haber, 2002; Haber and Calzavara, 2009), as well as mediating information flow between cortico-cortical structures via this transthalamic route (Guillery and Sherman, 2002; Sherman, 2005, 2007; Sherman and Guillery, 2005). Glutamate is the main neurotransmitter of communication between thalamus and cortex (Sherman, 2013).

**Figure 3:**
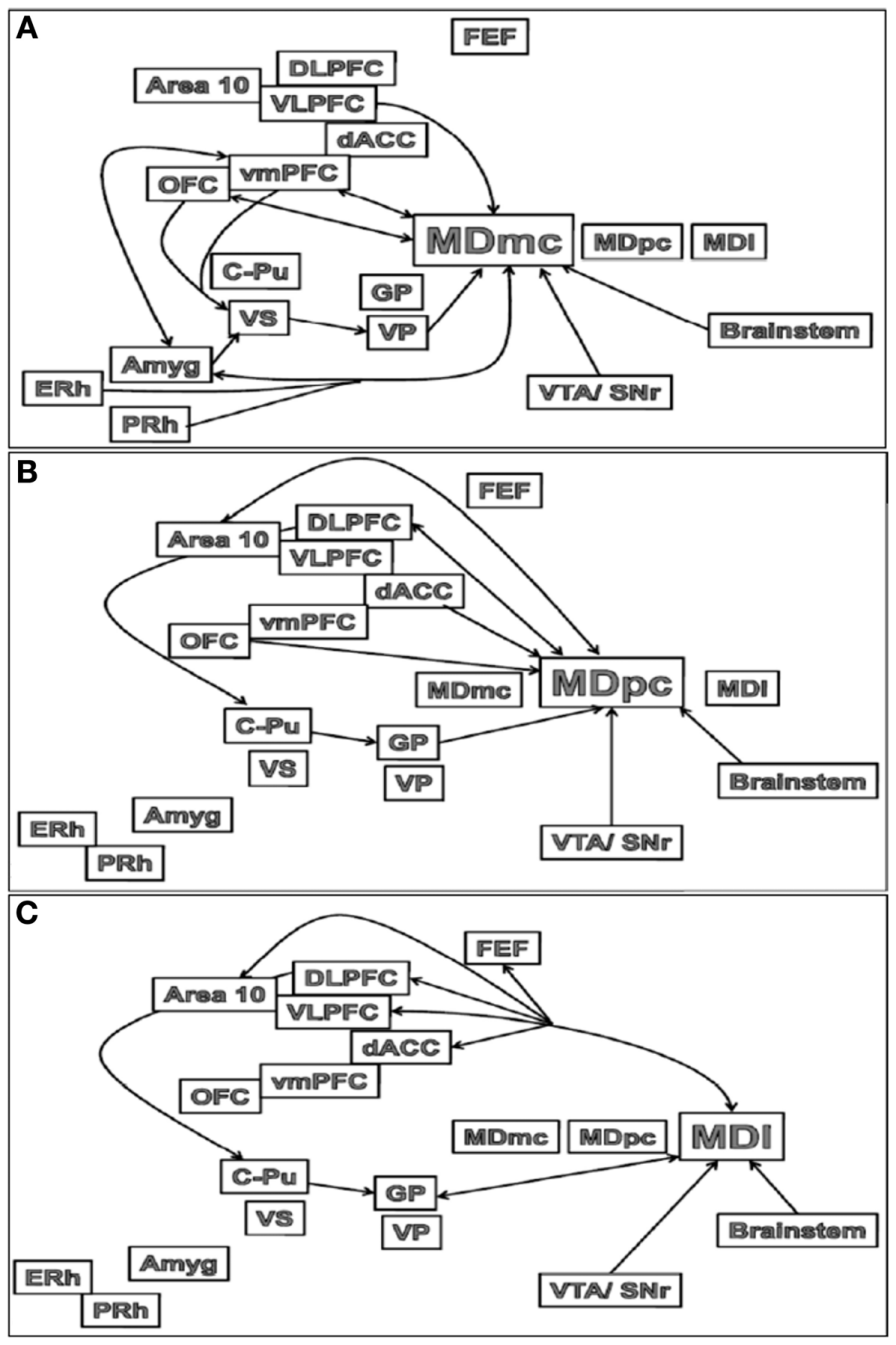
**Schematic illustrations of the main connections of the (A) MDmc, (B) MDpc and (C) MDl in the brain.** Abbreviations are provided in the text.

The major outputs of the MD are to the medial and lateral prefrontal and orbital frontal (OFC) cortices, and in some neuroanatomical tracing studies in rats, the medial PFC is said to be defined by the projections received from the MD nucleus (Groenewegen, 1988; Negyessy et al., 1998). Thus, these interconnections between the MD and PFC are segregated based on the subdivisions within the MD (see Figures 3A–C). The MDmc-PFC projections are almost exclusively reciprocal between the MDmc and the OFC and ventromedial PFC (vmPFC: areas 14, 25, 11, 13, and 12) but there is also a nonreciprocal input from ventrolateral PFC (VLPFC: area 45) and medial PFC (dACC: area 32 from the ventral and caudal aspects) (Preuss and Goldman-Rakic, 1987; Russchen et al., 1987; Barbas et al., 1991; Bachevalier et al., 1997; McFarland and Haber, 2002). Some of the midline nuclei [e.g., the intermediodorsal (IMD) and the paraventricular (PV) nucleus in rodents, see Figure 1] are also reciprocally connected to the OFC (Groenewegen, 1988). Thus, the MDmc and these midline nuclei have been regarded as a neuroanatomically functioning unit in rodents (Mitchell and Dalrymple-Alford, 2005). The MDpc has reciprocal connections with the dorsolateral PFC (DLPFC; areas 9 and 46) and area 10. There is also non-reciprocal inputs to MDpc from OFC (area 12, 13), VLPFC and the dACC (supracallosal area 24 and from the dorsal and rostral aspects of precallosal area 32 and 14) (Preuss and Goldman-Rakic, 1987; Russchen et al., 1987; Barbas et al., 1991; Bachevalier et al., 1997; Haber and McFarland, 2001; Erickson and Lewis, 2004). The most lateral parts of the MD that are combined with the ILn diffusely project to the PFC and dACC (supracallosal area 24) and exclusively to the frontal eye fields (FEF); the most prominent projection however is the topographically organized input to the basal ganglia (Preuss and Goldman-Rakic, 1987; Barbas et al., 1991; Bachevalier et al., 1997; Erickson and Lewis, 2004; Erickson et al., 2004).

#### Medial temporal lobes (MTL) afferents and efferents

In non-human primates there are connections from the association cortex of the temporal lobes [i.e., the entorhinal (ERh) and perirhinal (PRh) cortices] to the MDmc (see Figure 3A) and midline thalamic nuclei (Aggleton et al., 1986; Russchen et al., 1987; Saunders et al., 2005). These mainly course through the ventroamygdalofugal pathway and the inferior thalamic peduncle. In the rodent brain, it appears that only the rostral portion of area 35 of the perirhinal cortex projects to the MD (Burwell et al., 1995).

There are also amygdala (Amyg) projections to the MD (see Figure 3A). The central nucleus and basolateral nuclei project densely to the MDmc in monkeys and medial segment of MD in non-primates (Aggleton and Mishkin, 1984; Groenewegen et al., 1990; Krettek and Price, 1977). These projections from the amygdala to the medial MD are much sparser than the amygdala projections to the striatum and PFC (Jones, 1985). Krettek and Price (1977) reported that the fibers from the caudal part of amygdala terminate rostrally in medial MD and those from the rostral part terminate more caudally and ventrally in the medial MD. In turn, it has been documented in rodents that medial parts of MD project back to the basal grouping and anterior cortical nuclei of the amygdala, while the midline nuclei project to the central nucleus and the rostral part of the basolateral nucleus of the amygdala (Groenewegen et al., 1990).

In contrast, the central MDpc and more lateral parts of the MD (see Figures 3B,C) do not directly interact with the MTL and amygdala.

### Subcortical afferents and efferents

Some of the thalamic projections to the PFC represent in many instances the final link in fronto-striatal-thalamic circuits (Alexander et al., 1986; Groenewegen et al., 1990, 1999a; Haber and McFarland, 2001; Haber and Calzavara, 2009; Haber and Knutson, 2010). There are no neuroanatomical tracing studies that document direct projections from the caudate-putamen (C-Pu) to the medial thalamus in rats or in primates. Instead the C-Pu projects to the output structures of the pallidum via either direct or indirect pathways and then onto the medial and ventral thalamus. The direct pathway comprises of striatal projections to the internal segment of the globus pallidus (GP) and the reticular part of the substantia nigra (SNr: located in the midbrain) then to the thalamus. The indirect pathway comprises of striatal projections to the external segment of the GP, then to the subthalamic nucleus (STN), which in turn project to the internal segment of the GP and the SNr, and then to the thalamus^1^ (Haber et al., 1985; Groenewegen et al., 1990, 1993, 1997, 1999a,b; Tekin and Cummings, 2002; Haber and Calzavara, 2009). The internal segment of the GP projects predominantly to MDpc and MDl, while the ventral pallidum (VP) projects densely to MDmc. The most lateral subdivisions of the MD and many midline nuclei project to the basal ganglia (Berendse and Groenewegen, 1990; Groenewegen et al., 1990, 1999a; Gimenez-Amaya et al., 1995; Haber and McFarland, 2001; Haber and Calzavara, 2009).

From the brainstem in the rat, the locus coeruleus projects to all segments of the MD (Groenewegen, 1988). The median raphe projects most heavily to the MDl, whereas the dorsal raphe is strongly connected with the MDmc (Groenewegen, 1988).

The MDpc and MDl receive non-dopaminergic projections from the ventral tegmental area (VTA) and SNr (Groenewegen et al., 1990). The MDmc also receive projections from the VTA and SNr, but these projections are dopaminergic (Groenewegen, 1988). In addition, the reticular formation projects to all segments of the MD (Groenewegen, 1988).

A significant amount of thalamic neuromodulatory input is also received from the basal forebrain. Amongst many studies it is reported that the largest amount of basal forebrain inputs reaching the medial thalamus terminate in the reticular nucleus, with moderate terminal fields in the MDmc and sparse terminals in other sites (Hallanger et al., 1987; Groenewegen, 1988). These basal forebrain projections to the MD are predominantly GABAergic, while brainstem projections provide cholinergic inputs (Hallanger et al., 1987). For example, in cats, only 7–20% of basal forebrain neurons projecting to the MD are cholinergic (Bentivoglio et al., 1993).

Based on these differences in cortical-subcortical connectivity patterns among the MD, PFC, MTL, and basal ganglia, at least three separate MD thalamic neural circuits can be identified: a medial subdivision, including some of the midline nuclei and the MDmc, reciprocally connected to the OFC and vmPFC with further inputs from the VLPFC, rhinal cortex, amygdala, VS, and VP (Mitchell and Dalrymple-Alford, 2005); a central subdivision, the MDpc, reciprocally connected to the DLPFC and area 10 with further inputs from the OFC, dACC, dorsal striatum, and GP; and a lateral subdivision (Mitchell and Dalrymple-Alford, 2005; Lopez et al., 2009) including the intralaminar nuclei that is also interconnected to the dorsal striatum, GP and more diffusely to the PFC and FEF.

## Animal lesion studies

Studying memory and cognition with animal models is extremely insightful, in addition to being a useful way to overcome some of the limitations that are inherent in the clinical evidence. There are many advantages to developing animal models of memory processing. Surgical lesions in animals can normally be somewhat more circumscribed and involve subtotal, complete or even contra-lateral neuronal damage to connected structures. These planned lesions, if produced with a high degree of selectivity to the target structures of interest, can encourage a greater certainty about identifying the critical locus and also the particular kinds of memory deficits than are evident in comparative human cases. In addition, direct comparisons are possible between control and lesion animals, within pre- vs. post-operative testing or between subtotal lesions to one structure vs. another nearby structure.

Despite the benefits of experimental thalamic lesions, animal studies have, like the clinical evidence, also encountered difficulties and produced conflicting findings. This has resulted from the use of different techniques to create lesions in the MD, differences in the size and location of these lesions, and the extent of atrophy to surrounding target structures due to the inherent complexity of the medial and “non-specific” regions of the thalamus. Fortunately, the extent of brain damage in the medial thalamus has been minimized more recently by using neurotoxins that produce selective lesions to the individual structures that make up the medial diencephalon in animals. Thus, recent studies in rodents and non-human primates with very selective lesions to the mediodorsal thalamus using neurotoxins have been most insightful (Chudasama et al., 2001; Corbit et al., 2003; Mitchell and Dalrymple-Alford, 2005; Gibb et al., 2006; Mitchell et al., 2007a,b, 2008; Mitchell and Gaffan, 2008; Ostlund and Balleine, 2008; Pickens, 2008; Wolff et al., 2008; Lopez et al., 2009; Cross et al., 2012; Moreau et al., 2013).

Standardization of memory tasks and testing procedures for animals has also met with difficulties. Interpreting findings across studies and species can be problematic. Nevertheless, it is widely accepted that some cognitive tests provide adequate measures of animal memory that are analogous to human episodic recall tasks (Aggleton and Pearce, 2001). In addition, over the past few years, memory research has linked the work done in rats with the work of humans and non-human primates to a greater extent (Aggleton and Brown, 1999; Aggleton et al., 2000; Aggleton and Pearce, 2001; Morris, 2001; Uylings et al., 2003).

### Experimental mediodorsal thalamus lesions

Earlier work in animals focused on determining the one critical structure within the medial thalamus that was causing the memory deficits associated with thalamic amnesia. As mentioned, there are many candidates within the medial thalamus to fulfill this critical role. Neuropathological evidence reported in clinical cases of Wernicke-Korsakoff's syndrome supported a role for the MD in memory (Victor et al., 1971; Kopelman, 1995). However, Wernicke-Korsakoff's patients invariably suffer extensive neural damage due to the widespread effects of alcohol in the brain (Kril and Halliday, 1999), thus less equivocal evidence can only be obtained from experimental lesion studies involving circumscribed damage conducted in animal models. Table 1 details what we believe to be the extent of the experiments that have investigated cognitive and memory impairments after MD lesions over the past 40 years in rodents and monkeys collected using searches on pubmed involving mediodorsal, medial dorsal, dorsomedial, dorsalis medialis, and thalam^*^. Some of these studies and their conclusions are discussed below.

**Table 1:** **Summary of studies involving MD thalamic lesions assessing performance in an array of memory tasks over the past 40 years**.

**References**	**Lesion/Species/Type**	**Behavioral tasks**	**Training**	**Delay**	**Deficits reported**
Moreau et al., 2013	Lateral MD + ILn rats: NMDA	Spatial water maze	Post-op		No
		Visual water maze			No
Cross et al., 2012	MD rats: NMDA	Single item recognition	Post-op	5 m, 3 h	No
		Spatial location		5 m, 3 h	No
		Object-in-place		5 m, 3 h	Yes
		Recency memory		3 h	yes
Izquierdo and Murray, 2010	MDmc +Amyg + OFC macaques: NMDA	Reward devaluation	Post-op		Yes, neural circuitry important for reward based decision making
Chauveau et al., 2009	MD mice: ibotenic	Contextual serial discrimin	Post-op	24 h	With no stress MD only mildly impaired, with stress condition MD substantially impaired
		Retention with stress variable			
Dolleman-van der Weel et al., 2009	MD rats: NMDA	Morris water maze	Post-op		Transient deficit only
					Some impairments with strategy shifting
Lopez et al., 2009		Morris water maze	Post-op		No acquisition deficits, impaired in remote (25d) but not recent (5d) retrieval of correct quadrant
Mitchell et al., 2008	MDmc + Fx macaques: NMDA/ibotenic + ablation	300 OIP discriminations	Pre-op		Yes
		100 OIP discriminations	Post-op		Yes, combined lesions produced substantial new learning impairments
Mitchell and Gaffan, 2008	MDmc macaques: NMDA/Ibotenic	300 OIP discriminations	Pre-op		No
		100 OIP discriminations	Post-op		Yes, new learning impairments
Ostlund and Balleine, 2008	MD rats: NMDA	Instrumental conditioning	Pre-op		Yes, disrupted influence of Pavlovian cues over action selection, no impact on selection of actions based on expected value
Pickens, 2008	MD rats: NMDA	Pavlovian devaluation	Post-op		Impaired when switching from Pavlovian to operant contingencies but not when switching from one reinforcer to multiple reinforcer conditions
		Operant devaluation	Post-op		
		One vs. multiple reinforcers			
Wolff et al., 2008	Lateral MD + ILn Rats: NMDA	Allocentric spatial water maze	Post-op		No
		Egocentric spatial Y water maze			No
Block et al., 2007	MD rats:	Task set shifting T-maze			No, only impaired on new learning of strategies
Mitchell et al., 2007a	MDmc macaques: NMDA/ibotenic	Strategy implementation	Pre-op		No
		OIP association	Pre-op		Yes, new objects-in-place post-op
Mitchell et al., 2007b	MDmc macaques: NMDA/ibotenic	Reward devaluation	Post-op		Yes
Gibb et al., 2006	Lateral MD + ILn	Odor-place associations Odor discriminations	Post-op		Yes
	Rats: NMDA				No
		Place discriminations			No
Mitchell and Dalrymple-Alford, 2006	Lateral MD + ILn	Egocentric responding X-maze 8 arm radial maze	Pre-op		Impaired at matching body turn after delay
	rats: NMDA		Post-op		
					No
Chauveau et al., 2005	MD mice: ibotenic	Sequential alt	Post-op	5–30 s	Only impaired when delays mixed (30-5)
		Go/ No-go temporal alt		0–30 s	
					Impaired
Mitchell and Dalrymple-Alford, 2005	Medial MD; lateral MD + ILn	Radial maze	Post-op	2 h	No
		Go/No-go devaluation	Post-op		Yes, MDmc
	rats: NMDA	Single item (SOR)	Post-op		No
		Recency memory (TOM)	Post-op		Yes, MDmc and MDpc+ILn
Ridley et al., 2005	MD + IT marmosets: NMDA + ablation	Spatiovisual conditioning	Pre-op		Unilateral MD not impaired in retention. Combined crossed lesions caused mild impairments
		Visuospatial conditioning retention and learning	Post-op		
Corbit et al., 2003	MD rats: NMDA	Instrumental conditioning	Post-op		MD acquired conditioning then deficits in selective devaluation effect during extinction
		Devaluation extinction tests			
Ridley et al., 2002	MD+AT marmosets: NMDA	Visuospatial conditional task	Pre-op		Combined MD+AT impaired in retention but separate MD or AT lesions were not
		Visuovisual conditional	Post-op		
		Concurrent discriminations			
					No
					No
Alexinsky, 2001	MD rats: ibotenic, excision	3/8 baited radial maze	Pre-op		MD = less correct visits only;
		New Route—Pre-exp- Y/N			Pre-exposure –Y = MD deficits;
		Contextual light change			
					MD adapted
Chudasama et al., 2001	MD rats: NMDA	Visual discriminations and reversals with touch-screen	Pre-op		MD = impaired at reversal of all three visual discriminations
			Post-op		
Gaffan and Parker, 2000	MDmc macaques: aspiration	Visual scene memory	Pre-op		Yes
		Object-reward associations	Pre-op		Retention = No
					New Post-op Learning = Yes
Floresco et al., 1999	MD rats: bilateral lidocaine infusion	Delayed radial maze	Post-op	30 min	Pre-test infusion severe deficits.
		Non delayed random foraging radial maze	Post-op		
					Not impaired.
		Delayed radial maze and Pre-test infusion only	Post-op	30 min	MD/N Acc. not impaired. A PL/N Acc. group were also impaired
Kornecook et al., 1999	MD rats: electrode	Visual object discrimination	Pre-op		No deficits on retention of discriminations learnt pre-op up to 58 days prior to surgery
			Post-op		
					No
Zhang et al., 1998	MD rats: NMDA	Go/no-go DNMTS odors	Pre-op	4–20 s	MD mild and transient deficits;
		Olfactory discrimination			
					No
Burk and Mair, 1998	MD rats: NMDA	Place DMTS, operant boxes	Pre-op	1–13 s	No
		Serial reversal learning	Post-op		No
Hunt and Aggleton, 1998a,b	MD rats: NMDA	Standard radial maze	Post-op	60 s	No
		Radial maze (45° rotation)	Post-op	60 s	Yes
		T-maze Alt		10 s	No
		8-arm radial maze		15, 60 min	Yes, exacerbated by AT damage
		SOR			
					No
Hunt and Aggleton, 1998a,b	MD rats: NMDA	8-arm radial maze CCP	Post-op	10–40 s	No
		Exploratory Activity			No
		T-Maze MTP			Yes, slower to acquired task but no delay deficits
		T-Maze Reversal			No, MD more perseverative errors than controls
Parker et al., 1997	MD macaques: ablations	DMTS	Pre-op	0–30 s	Yes for large stimulus set size but not small set size
		Concurrent discriminations	Post-op		
		Rule reversal learning	Post-op		No
					No
Peinado-Manzano and Pozo-Garcia, 1996	MD rats	Delayed alternation in operant boxes	Pre-op	0–80 s	Moderate and transient impairment for 0–40 s and severe impairment for 80 s
Young et al., 1996	MD rats: RF	DNMTS in operant boxes	Post-op	1.8–8.8 s	MD produced deficits in acquisition of the radial maze task
		8-arm radial maze			
Krazem et al., 1995	MD mice: ibotenic	T-Maze Spatial repetition	Post-op	5 min, 24 h	No
		T-Maze Reversal			Yes, MD required more trials
Hunt et al., 1994	MD rats: NMDA	Object, concurrent and configural discrim	Post-op		MD mildly impaired on concurrent discriminations
Gaffan et al., 1993	MD + Amyg + VMPFC macaques: ablation	2-choice visual discrim task with food reward for correct choices	Post-op		Crossed lesions caused severe deficits in post-op acquisition
Mumby et al., 1993	MD rats: electrolytic	Visual object recognition DNMS	Post-op	4 s acq.	Yes, more trials to learn, then delay dependent deficits
			Pre-op	4–300	
					30–300 s
					Yes, more trials to reacquire
Neave et al., 1993	MD rats: NMDA	DNMTP	Post-op	0–32 s	No
		Spatial discrim and Reversal			No
Gaffan and Watkins, 1991	MD macaques: ablation	Learning of visual stimuli associated with different amounts of food	Pre-op		Yes, impaired on retention of pre-op reward stimuli associations and impaired in new learning of further reward stimuli associations
			Post-op		
Hunt and Aggleton, 1991	MD rats: RF, ibotenic	Y-Maze Object recognition	Post-op	0–60 s	Yes
		T-Maze Delay alt		10–60 s	Yes, spatial memory deficits only a consequence of anterior thalamic involvement
M'Harzi et al., 1991	MD rats: electrolytic	Radial maze	Post-op		Yes
		Place recognition			No
		Object recognition			No
Peinado-Manzano and Pozo-Garcia, 1991	MD rats: electrolytic	Operant delay alt	Post-op	0–80 s	Yes
Gaffan and Murray, 1990	MD + Amyg + vmPFC macaques: ablation	2-choice visual discrim with food reward for correct choices	Post-op		Bilateral lesions to MD impaired
					Crossed unilateral lesions not as impaired as bilateral lesions to any of the single regions.
Stokes and Best, 1990a	MD rats: electrolytic	8-arm radial maze	Post-op		Yes, combined MD and AT damage
Stokes and Best, 1990b	MD rats: ibotenic	8-arm radial maze	Post-op		Yes, combined MD and AT damage
Winocur, 1990	MD rats: electrolytic	Memory for food preferences	Post-op	0–8 d	No
			Pre-op		Yes, only if no delay btw acquisition and surgery Not impaired with 2 d between acquisition and surgery
Beracochea et al., 1989	MD rats: ibotenic	8-arm radial maze	Post-op	15, 45 s	No
		T-Maze temp alt			Yes = 15 s but not with 45 s delay
		T-Maze spatial reversal			
					No
Stokes and Best, 1988	MD rats: electrolytic	8-arm radial maze	Pre-op	0 s	Yes, combined MD and AT damage
Zola-Morgan and Squire, 1985	Posterior MD macaques: electrolytic	Visual DNMTS	Post-op	8–60 s, 10 min	Yes, delay independent
		Pattern discrimination			No, analogous to preserved capacity for skill learning in human amnesic patients
Winocur, 1985	MD rats: electrolytic	Delayed alternation	Post-op	0–21 d	Yes, impaired acquisition and impaired at all delays
		Passive avoidance			
					No
Aggleton and Mishkin, 1983a	MD macaques: ablation	Object recognition	Post-op	120 s	Yes
		Object-reward associations			Yes
Aggleton and Mishkin, 1983b	MD +AT macaques: ablation	Object recognition	Post-op	120 s	Yes
		Visual pattern discrim			No
		Spatial delayed response			No
Isseroff et al., 1982	MD macaques: RF	Spatial delayed response	Post-op	5 s	Yes
		Visual pattern discrim			No
		Delayed alternation			Yes
		Object discrim + reversals			No

Abbreviations *Alt* *alternation* *Discrim* *discrimination* *DNMTP* *delayed non match to position* *DNMTS* *delayed non match to sample* *Egocentric* *egocentric discrimination* *FX* *fornix* *RF* *radiofrequency* *Seq* *sequential* *SOR* *spontaneous object recognition* *post-op* *post-operative* *pre-op* *pre-operative*.

*For other abbreviations see elsewhere in the text*.

#### Non-human primates

Monkey studies have demonstrated that aspiration lesions to the MD (i.e., typically including the magnocellular and the parvocellular subdivisions and other medial thalamic structures as well as potential fibers of passage passing through this region) cause impairments in recognition memory, deficits in new learning of object-in-place (OIP) discriminations and object-reward associations. These lesions also produce impaired performance in the spatial delayed alternation task and delayed response task but not in object reversal (associative memory task) and visual pattern discrimination (Isseroff et al., 1982; Aggleton and Mishkin, 1983a,b; Zola-Morgan and Squire, 1985; Gaffan and Murray, 1990; Gaffan and Watkins, 1991; Parker et al., 1997; Gaffan and Parker, 2000). Other studies have also highlighted how interactions between interconnected structures of the amygdala, vmPFC and MD are important for postoperative new learning of two-choice visual discriminations associated with differing amounts of food reward (Gaffan et al., 1993). Despite this extensive range of deficits linked to damage in the MD, it did not appear that MD lesions by themselves were the critical source of dense amnesia linked to cases of thalamic and diencephalic amnesia suffered in patients. For example, Parker et al. (1997) found that bilateral ablations to MDmc did not produce recognition memory deficits as severe as those reported after bilateral perirhinal cortex ablations, and the animals were also not as markedly impaired as amnesic patients with recognition memory deficits (Aggleton and Shaw, 1996).

Parker and Gaffan (1998) proposed that ablation of the MDmc in primates produces hypoactivity in the PFC and, therefore, the deficits in cognitive testing after MD lesions might be ascribed to frontal dysfunction. Given the extent of dense reciprocal connections between the MD and PFC, it makes sense to propose that damage to the MD may result in dysfunction within the PFC and that this disruption causes deficits in cognition and memory (Isseroff et al., 1982). The PFC is associated with higher order cognitive functioning, often labeled “executive functioning” in humans. It has been suggested that lesions to the MD could disrupt pathways leading to the PFC and may affect processes that are typically governed by the PFC, including attention, inhibition, planning, coordination, and strategy selection, which could then produce memory impairments on tasks (Gaffan and Parker, 2000). Certainly all of the above tasks that produce deficits after MD lesions are also sensitive to damage in the PFC (Fuster, 2008; Chudasama, 2011).

More recently, selective neurotoxic lesions to the MDmc have confirmed the importance of this medial subdivision in new learning of OIP discriminations and in a reward satiety devaluation task, as neurotoxic lesions of the MDmc produce impaired performance on these tasks (Mitchell et al., 2007a,b, 2008; Izquierdo and Murray, 2010). However, the same selective lesions to MDmc do not impair the retention of pre-operatively acquired information (Mitchell et al., 2007a; Mitchell and Gaffan, 2008). One such task that assesses retention of pre-operative information is the strategy implementation task (Gaffan et al., 2002). In this task, animals learn a specific strategy for responding to objects presented on a touchscreen in order to receive reward. Pre-operative performance for individual animals is compared with post-operative performance. Animals with crossed unilateral lesions that disconnect the whole of PFC in one hemisphere from inferotemporal cortex in the contralateral hemisphere (PFC × IT) cause impairments on this task (Gaffan et al., 2002) as do bilateral ablations to the VLPFC (Baxter et al., 2009). However, damage to the MDmc did not disrupt the animals' ability to implement the strategy that they had acquired pre-operatively (Mitchell et al., 2007a), despite the strong reciprocal interconnections with PFC and input from IT.

**Figure 4:**
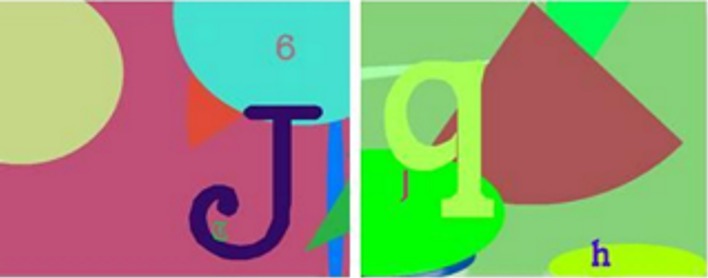
**Two examples of object-in-place (OIP) discrimination problems.** Each discrimination problem had two different “objects” (one rewarded and one non-rewarded) embedded within a unique colored and patterned background akin to a “scene”; the objects are the differently colored typographic characters “B” and “m” in the left panel and “J” and “h” in the right panel.

In the same study, another task, the OIP discrimination learning task, was also learnt pre-operatively although in this task animals learn 20 new pairs of OIP discriminations (see Figure 4 for example stimuli of the OIP discriminations) during each session across eight concurrent repetitions of the set of 20 pairs of OIP discriminations. To assess impairments in this task, the performance of each animal is compared during a pre-operative test of 10 sessions and compared, after recovery from neurosurgery, to a post-operative test of 10 sessions. The neurotoxic MDmc lesions caused animals to make more errors during postoperative testing, so it could be concluded that deficits were linked to new learning of information as opposed to the retention of specific information acquired pre-operatively (Mitchell et al., 2007a). Critically though, this evidence demonstrated that the damage to the MDmc was not simply causing widespread PFC dysfunction to account for the observed cognitive deficits. Furthermore, the extent of the deficits on the OIP task were similar across two different lesion studies provided convincing evidence that the neurotoxin lesion technique used by Mitchell et al. (2007a) worked as effectively as the previously used ablation method (Gaffan and Parker, 2000).

In addition, the types of errors made in learning the OIP discriminations produced after bilateral MDmc lesions are not suggestive of problems with perseverative responding during learning (Mitchell et al., 2007a). However, bilateral ablations to the VLPFC do produce perseverative responding during new learning of OIP discriminations (Baxter et al., 2008). This evidence further confirms that damage to the MDmc does not simply produce a generalized impairment in memory by causing dysfunction of prefrontal functioning as had been previously proposed.

However, other studies (e.g., Parker et al., 1997) have shown that damage to the MD does not impair new learning or retention on recognition memory tasks when the stimulus set size is small. So it could also be argued that as the stimulus set size (four pairs of objects) is small in the strategy implementation task, no further learning is occurring. This theory was further tested (Mitchell and Gaffan, 2008) by comparing retrograde amnesia and anterograde amnesia within the same animals using the same types of OIP discrimination stimuli for both types of memory (see Figure 4) from a large sample size of 400 pairs of discriminations. In addition, a one-trial retention test was used to assess memory retention; this test is a pure measure of postoperative retention, uncontaminated by post-operative re-learning (Dean and Weiskrantz, 1974). Interestingly, damage to the MDmc using neurotoxins caused no impairment in the one-trial postoperative retention test. That is, the monkeys with bilateral MDmc neurotoxic lesions showed good retention (i.e., no retrograde amnesia) of the 300 pairs of OIP discriminations that they had acquired pre-operatively, when the errors made during their pre-operative retention test were compared with errors made during their post-operative retention test (Mitchell and Gaffan, 2008). In contrast, the same animals were markedly impaired in new postoperative learning (anterograde amnesia) of a further set of 100 novel pairs of OIP discriminations presented concurrently across sessions (Mitchell and Gaffan, 2008). It was concluded from this evidence that the MDmc is critical for the processing of new information more so than in the retention of information acquired prior to damage.

Further research from our laboratory has extended our understanding about some of the brain regions involved in retrograde amnesia and anterograde amnesia using this OIP retention and new learning task. The effects of lesions to different subcortical and cortical structures have been assessed (Mitchell et al., 2008; Mitchell and Buckley, submitted). One such study assessed the effects of combining neurotoxic MDmc lesions with bilateral fornix transection [a lesion that produces more widespread brain damage to medial diencephalic structures as well as disrupting interconnections with the PFC and medial temporal lobes (Mitchell et al., 2008)]. Interestingly these combined lesions produced dense amnesia for new learning as well as retention, yet still they confirmed that subcortical damage produces more severe deficits in anterograde amnesia than in retrograde amnesia.

Clearly animals with bilateral MDmc lesions have provided greater understanding of the critical role that the MDmc plays in differing forms of memory processing. Animals with MDmc lesions have also been assessed on tasks investigating other cognitive processes e.g., reward (satiety) devaluation. For example, animals with selective bilateral neurotoxic lesions to the MDmc are also impaired on a computerized version of a classic food satiety devaluation task (Malkova et al., 1997) demonstrating the importance of the MDmc within the neural circuit crucial for reward devaluation (Mitchell et al., 2007b) that also includes the OFC and amygdala (Malkova et al., 1997; Baxter et al., 2000; Izquierdo and Murray, 2010). Interestingly though, lesions to MDmc did not impair the animals' ability to learn the 60 pairs of object-reward associations presented concurrently over successive sessions during initial postoperative acquisition training before performing the reward satiety devaluation testing in this task. In this reward devaluation paradigm, the monkeys must first learn postoperatively to link one of the two pairs of objects in each presentation with a peanut or chocolate candy reward, 50% of one of the stimuli from the pairs of objects was rewarded for a correct choice with a peanut (while the other object was not associated with any food) and the other 50% of the pairs of objects with a chocolate candy. Presumably this lack of deficit in new learning during the initial acquisition was linked to the smaller set size of stimuli (Parker et al., 1997) as well as to the salience of the rewards [e.g., bilateral lesions to the OFC and the amygdala also do not impair the initial postoperative acquisition of the object-reward associations in this task (Malkova et al., 1997; Baxter et al., 2000)]. Other studies though have shown that bilateral MD lesions do impair the ability of the animals to associate pairs of stimuli with differing amounts of food rewards (Gaffan and Watkins, 1991).

In contrast, MD lesions do produce deficits in new learning of larger sized samples of concurrent object-reward association problems over sessions, although the lesion does not impair the retention of pre-operatively acquired object-reward associations (Gaffan and Parker, 2000). Damage to parts of the PFC (e.g., crossed unilateral disconnection of PFC × IT lesions) does not impair concurrent learning of visual object discriminations, where the animal learns to associate visually presented objects with food (or no) reward and the presentation of each object pair is separated in time by presentation of other pairs of objects (Gaffan et al., 2002). Interestingly, this same PFC × IT disconnection lesion does, however, severely impair serial learning of visual discriminations, where the animal learns to associate single pairs of objects with food (or no) reward and the presentation of each object pair occurs immediately after one another (Browning et al., 2007). Concurrent object-reward association learning is qualitatively different to learning serial presentations of visual discriminations (Murray and Gaffan, 2006). It remains to be fully tested whether bilateral MD lesions produce dissociable deficits in learning concurrent vs. serial visual discriminations, although as previously shown MD lesions produce deficits in within-session new learning of OIP discriminations (Gaffan and Parker, 2000; Mitchell et al., 2007a).

#### Rodents

In rats, many studies assess the rats' ability to forage for food using T-mazes, water mazes and radial arm mazes, taking advantage of their natural curiosity to explore novel environments for food. Many strategies can be used by the animals to complete these tasks successfully (Dudchenko, 2001). One strategy involves spatial navigation based on the use of extra maze cues (e.g., door, windows, lights, posters, experimenter, etc.) within the testing environment (spatial cues) to help guide their optimal exploration and ensure they do not return to the same location twice. Animals with selective neurotoxic lesions to the MD show comparable performance to control animals when they use spatial cues to guide their navigation in radial arm maze tasks (Beracochea et al., 1989; Hunt and Aggleton, 1998a; Mitchell and Dalrymple-Alford, 2005), unless they incorporate a delay (Harrison and Mair, 1996; Floresco et al., 1999) or produce more widespread damage that also includes the AT (Stokes and Best, 1988, 1990a,b; Hunt and Aggleton, 1991, 1998a). It has been proposed that MD deficits in delay tasks are presumably a consequence of widespread disruption to PFC functioning (Hunt and Aggleton, 1998b). Floresco et al. (1999) contrasted working memory performance using a spatial delayed responding task and non-delayed spatial tasks to show that the interaction between the PFC and the MD mediates “context-dependent retrieval and manipulation of recently acquired information.” Furthermore, this study provided evidence, via lidocaine infusions into the MD, to show that MD alone is not sufficient to affect episodic-like memory processing on spatial memory tasks (Floresco et al., 1999). Instead, it is widely accepted that spatial memory processing deficits related to the medial thalamus are governed by the anterior thalamic nuclei and their interconnections to the extended hippocampal system, also known as the Delay-Bryon neural circuit (Aggleton and Brown, 1999).

Other researchers have observed in rats with bilateral MD lesions certain behavioral deficits that could result in memory impairments, for example, an inability to adopt different strategies, or changes in activity and exploration levels or deficits in withholding spatial responses (Hunt and Aggleton, 1998a,b; Floresco et al., 1999; Block et al., 2007; Ostlund and Balleine, 2008). All of these types of deficits are also observed in rats with damage to regions of the PFC (Chudasama, 2011).

Rodent studies have been instrumental in demonstrating the distinct, interdependent involvement of adjacent medial thalamic structures in memory and other cognitive deficits. Dissociable deficits between the MD and adjacent anterior thalamus (AT) have been reported (Chudasama and Muir, 2001; Chudasama et al., 2001; Corbit et al., 2003; Mitchell and Dalrymple-Alford, 2005, 2006). Corbit et al. (2003) assessed the effects of highly selective MD and AT lesions in rats on instrumental conditioning. Rats with either MD or AT lesions were both able to acquire the instrumental performance but during the degradation of the action-outcome contingency test, the rats with MD lesion were unable to demonstrate reliable devaluation effects. This deficit shown by the rats with MD lesions was distinct from the rats with AT lesions and controls, which did not differ, and suggests that the MD contributes to deficits in encoding and/or utilizing the action-outcome association (Corbit et al., 2003). Mitchell and Dalrymple-Alford (2005) have also demonstrated dissociable impairments in rats with lesions of the medial MD compared to the lateral MD or to the AT on various cognitive tests. The damage to the medial MD impairs go/no-go reward value discriminations and recency memory with a 2-h delay, but had no impact on spatial memory processing using an 8-arm radial maze or spontaneous object recognition (SOR) memory (see below). Lateral MD lesions produced mild deficits in 8-arm radial maze performance and recency memory but had no impact on go/no-go reward value discriminations or SOR memory (see below). In contrast, AT lesions produced deficits on 8-arm radial maze performance but they had no effect on recency memory using a 2-h delay. Interestingly, AT lesions do impair the ability to remember the pseudorandom order of six odors (Wolff et al., 2006). This deficit may be linked to the nature of the associative memory processing involving reward that is required in this particular task but that is not present in the spontaneous exploration paradigm used in the recency memory task (see below). Further dissociations in performance have been reported in rats with lesions to the lateral MD or to the AT, with only damage to the lateral MD impairing the rats' memory in a delayed-match-to-sample task using an egocentric (body-turn) response in a cross-maze; damage to the AT left performance intact (Mitchell and Dalrymple-Alford, 2006). Further studies from the same laboratory have provided more insight into the dissociable effects of lesions to the AT and lateral MD combined with ILN lesions in learning and memory processing (Gibb et al., 2006; Wolff et al., 2008; Lopez et al., 2009; Moreau et al., 2013). These authors concluded from this and the above evidence that no single medial thalamic structure is critical for all of the memory and other cognitive deficits associated with thalamic amnesia. Instead many subdivisions of medial thalamic nuclei are contributing to independent neural networks via subcortical and cortical interactions and are integrating information for successful cognition (Mitchell and Dalrymple-Alford, 2005, 2006). Other research (e.g., Eleore et al., 2011) has also documented similar roles for other thalamic nuclei, namely the reuniens, in supporting the acquisition of associative learning using a classical eyeblink conditioning task with a trace paradigm, because high frequency train stimulation directed at the reuniens in behaving mice prevented the proper acquisition of the task.

The experiment demonstrating bilateral MD involvement in recency memory (Mitchell and Dalrymple-Alford, 2005) has been further confirmed by Cross et al. (2012). The medial PFC (mPFC) is involved in recency memory processing in rodents (Mitchell and Laiacona, 1998; Hannesson et al., 2004; Cross et al., 2012) and patients with Korsakoff's syndrome and frontal lobe damage have problems with temporal processing and recency discriminations (Kopelman et al., 1997; Kopelman, 2002; Fuster, 2008).

Cross et al. (2012) have demonstrated in rodents the importance of neural communication in MD-mPFC circuitry for successful recency recognition memory. After combining crossed unilateral lesions of the MD and mPFC, a lesion that disconnects the structures in both hemispheres, animals were impaired in the recency recognition memory task. In contrast, after a combined ipsilateral unilateral lesion of the MD and mPFC (essentially a control lesion of these two structures that leaves one hemisphere functioning) recency recognition memory performance was left intact. The authors proposed that “during associative or recency recognition memory tasks, the MD–mPFC connection might be necessary to direct ongoing behavior toward, for example, the novel object–place configuration” (Cross et al., 2012). This study highlights how the interplay of communication within and between MD-mPFC networks is clearly critical for cognition.

After neurotoxic lesions to MD, rodents are not impaired at SOR tasks (Hunt and Aggleton, 1998a; Mitchell and Dalrymple-Alford, 2005; Cross et al., 2012). As already noted above, conflicting evidence exists for the role of MD in various recognition memory tests. Several studies have reported deficits in object recognition using rats involving delay non-matching-to-sample tasks that involve object-reward associations (Hunt and Aggleton, 1991; Mumby et al., 1993), while others have reported no deficits on various unrewarded recognition memory tasks that rely on spontaneous exploration instead (M'Harzi et al., 1991; Hunt and Aggleton, 1998b; Kornecook et al., 1999; Mitchell and Dalrymple-Alford, 2005). The most parsimonious explanation is that the task demands related to reward are different, as SOR does not involve reward but rather relies on spontaneous exploration while delayed-matching or non-matching to sample tasks normally reward the animal for a correct response, thus engaging associative memory networks instead (Parker et al., 1997; Gaffan and Parker, 2000). In addition, it is now known that pre-operative training is a critical factor in learning and memory tasks, as damage to the MD does not impair retention of pre-operatively acquired information associating objects and rewards (Gaffan and Parker, 2000; Mitchell and Gaffan, 2008). Also, there may be cross species differences in neuroanatomy. That is, in macaques there is a distinct projection from the rhinal cortices (perirhinal and entorhinal) to the MDmc (Aggleton et al., 1986; Saunders et al., 2005), while a similar projection is not as robust in rodents (Burwell et al., 1995).

As in monkey studies, researchers have investigated the devaluation effects after bilateral MD lesions in rodents. Pickens (2008) has systematically assessed rats with MD lesions on many variants of devaluation testing using Pavlovian and operant contingencies, and single and multiple reinforcement paradigms. Pickens concluded from this series of experiments, that the MD is important in devaluation circuits only “in cases in which previous associations need to be suppressed in order for new associations to be learned and control behavior, otherwise the devaluation circuit does not require MD” (Pickens, 2008).

Thus, through experimental testing in both rats and non-human primates it has been shown that the different subdivisions of the MD provide critical contributions to successful cognitive processing in many different tasks. Principally, the MD in conjunction with its neuroanatomical connections is important for some forms of recognition memory, recency memory processing, and further prospective integration of the rewards associated with successful responses to govern additional responses, as well as new learning of OIP discriminations, but not their retention. The subdivisions of the MD provide key roles in helping integrate object/reward/response information for successful new learning and successful additional (future) responding. Furthermore, and most importantly, it has been demonstrated that the MD contributes to successful cognition, rather than causing memory and other cognitive deficits by simply causing a generalized dysfunction of the PFC.

## Electrophysiology

A recent review of single unit recordings in macaques (Watanabe and Funahashi, 2012) provides insight into how the MD contributes to successful performance during working memory (delayed oculomotor response) tasks. The review highlights clear interplay between the MD and PFC, as suggested by other studies. For example, neurons in the MD have shown cue-, delay- and response-period activity, similar to the discharge patterns observed in DLPFC, although most neurons exhibited sustained excitatory response during the delay period (Tanibuchi and Goldman-Rakic, 2005; Sommer and Wurtz, 2006; Watanabe and Funahashi, 2012). One study (Alexander and Fuster, 1973) in particular showed attenuation in magnitude of the delay-period response following cooling of the DLPFC suggesting that the projection neurons of PFC control task-related activity of the MD.

Further experiments have shown that the MD seems to contribute to prospective encoding more so than DLPFC during the delay period (Funahashi et al., 2004; Watanabe and Funahashi, 2012). Watanabe and Funahashi (2012) have proposed that the MD is the major area that provides information regarding impending behavior to the DLPFC. In contrast, retrospective sensory information is maintained during the delay period in the DLPFC and this could play an important role in helping to generate prospective motor information (Watanabe and Funahashi, 2012). The response-period active neurons were more frequent in MD than in DLPFC reflecting a bias toward processing motor aspects of the task by these thalamic nuclei, confirmed further by population vector analyses (Watanabe and Funahashi, 2012).

Other electrophysiology studies have shown that the MDmc of primates contain neuronal populations that signal information concerning prior stimulus occurrence (Fahy et al., 1993), that is linked to interconnected regions of the medial PFC and the perirhinal cortex (Brown and Xiang, 1998; Xiang and Brown, 2004), although the role of the MD within this neural circuitry is still uncertain.

Finally, another study has used single unit recording to demonstrate how the PFC and MD interact in cognitive tasks. Recent work by Kellendonk and colleagues (Parnaudeau et al., 2013) using a mouse model of cognitive deficits in schizophrenia has shown a subtle decrease in MD activity to disrupt the thalamic-PFC neural circuitry and cognition. They recorded single units in MD neurons during choice phase vs. reward phase of the T-maze task and demonstrated decreased MD activity interfered with task-dependent modulation of MD-PFC synchrony, which correlated with the cognitive deficits of the mice.

## Theories about MD involvement in memory processing

Aggleton and Brown (1999) suggested that the MD, and the perirhinal cortex of the medial temporal lobe, may play a role in a system responsible for familiarity-based recognition processes. However, this proposal remains debated because the direct neural connections between the MD and perirhinal cortex are sparse and clinical evidence (Pergola et al., 2012) and animal lesion evidence (as detailed above) remains equivocal. While it is widely accepted that the perirhinal cortex contributes to recognition memory, the contribution attributed to the MD remains uncertain. The evidence in the clinical cases of deficits in recognition memory following damage in the MD is mixed (Cipolotti et al., 2008) with some researchers reporting no such impairments (Shuren et al., 1997; Edelstyn et al., 2002). However, given that the majority of evidence supports the MD being involved in memory, and that its role is not just confined to familiarity judgments, further models of MD functioning in memory processes are required.

Other researchers have proposed that the MD has a deferential role in memory processing caused by disruptions in executive functioning which is processed by the PFC. It has been suggested that the memory impairments resulting from lesions to the MD are secondary to the primary disruptions in executive functioning, e.g., deficits in attention or withholding responses/inhibition and perseverative responding in both humans and animals (Zola-Morgan and Squire, 1985; Hunt and Aggleton, 1998b; Floresco et al., 1999; Van der Werf et al., 2000; Schmahmann, 2003).

Van der Werf et al. (2003b) in a review of clinical evidence suggest that the AT and MD each has a functional role in declarative memory processes. The authors propose that the different nuclei of the thalamus play different roles at varying levels of declarative memory functioning, namely the AT and MD are involved in processing the contents of the stimuli for storage and recall. The AT influences the selection of material to be stored and remembered, whereas the MD is involved in the coordination and selection of strategies used to retrieve material. The intralaminar and midline nuclei maintain a necessary state of arousal amongst the cortical regions involved in the ongoing memory processes. These groupings of nuclei then work in parallel to mediate and allow memory functioning.

In contrast to these proposals, Gaffan, Mitchell and colleagues have proposed that the MD, in particular MDmc has an important integrative role in conjunction with the PFC in episodic-like declarative memory, due to the prominent interconnections among these structures (Gaffan and Parker, 2000). The MDmc has a specific role in supporting new learning of information, contributing to the successful acquisition rather than the retention of previously acquired information (Mitchell et al., 2007a, 2008; Mitchell and Gaffan, 2008). As highlighted above, the MD plays a key role in helping integrate object/reward/response information for successful new learning and successful additional (future) responding. Furthermore, Mitchell and colleagues have suggested that the role of MD in learning and memory is not simply a consequence of causing generalized disruption to PFC functioning (Mitchell et al., 2007a; Mitchell and Gaffan, 2008).

Aggleton et al. (2011) have revised their model of MD involvement in recognition memory. Their latest model, the multi-effect multi-nuclei model, asserts that the MD can contribute to both familiarity and recollective processes either directly via an interaction with the PFC or indirectly as a result of cortical diaschisis (Aggleton et al., 2011). This model is supported by recent findings regarding associative recognition (Cross et al., 2012), along with recent clinical results (Pergola et al., 2012) that point to contributions from the parvocellular MD for recollective aspects of recognition.

## Re-evaluating mediodorsal thalamus in memory and where to from here

As indicated from the above survey of the contribution of the MD to specific forms of memory and decision-making, some conclusions have been drawn but much debate remains. Nevertheless, the evidence thus far provides some understanding and certainly helps with future directions. Thus, the animal evidence (and also the clinical evidence although not reviewed here) simply doesn't support the notion that there is a single structure within the medial diencephalon that is responsible for the extent of anterograde and retrograde memory deficits associated with diencephalic (or thalamic) amnesia. Furthermore, given the extent of variability in other cognitive deficits observed after damage to the MD it is not possible that one specific structure or subdivision of the MD is the critical locus of these deficits. Instead, the evidence suggests that the subdivisions of the MD, and subdivisions of other medial thalamic structures, are each functioning within independent but integrated neural circuits, all of which are important for specific aspects of cognitive processing, and together they form a group of critical networks in the brain that are important for learning and memory as well as many other forms of cognition.

The current evidence points to the role of higher order thalamic structures, in our case the MD, in mediating the complex functioning within the PFC, via the transthalamic route (Sherman and Guillery, 2002). Neuroanatomical tracing studies have positioned the various subdivisions of the MD within separate but integrated neural circuits based on their respective interconnections. Moreover, as reviewed here, animal models of complete bilateral lesions to the MD as well as more selective lesions to individual subgroupings of the MD (i.e., medial MD, central MD and lateral MD) have demonstrated deficits in various tasks that assess new learning, recognition memory associated with reward, reward devaluation and recency memory processing, but not retention of previously acquired information. Manifestations of such deficits are often similar, but often can also be dissimilar to deficits seen after damage within the PFC (Fuster, 2008; Chudasama, 2011).

Thus, it may be proposed that the transthalamic connections linking the MD to the cognitive PFC are more important for supporting the learning of new information than for retention of previously acquired information (Mitchell et al., 2007a; Mitchell and Gaffan, 2008), perhaps by way of regulating cortical synchrony between regions of the PFC and MTL that support acquisition of new information. Others (Saalmann et al., 2012) have demonstrated how the pulvinar (another higher order thalamic relay structure) regulates cortico-cortical communication based on attention demands. This group combined simultaneous neural recordings in the pulvinar, V4 and area TEO (in the medial temporal lobes) while monkeys performed a visuospatial attention task. Precise interconnected target regions were identified via diffusion tensor imaging (DTI). The findings showed that the pulvinar regulates cortical synchrony between these connected structures according to the attentional allocation of the task (Saalmann et al., 2012).

In contrast to deficits in new learning, the evidence suggests that cortical structures are more important for the retention of information learnt prior to brain injury (retrograde amnesia). Impairments in retention are reported after restricted damage to selective cortical structures highlighting how some of these cortical regions are more important for memory of previously acquired information (Dean and Weiskrantz, 1974; Thornton et al., 1997; Mitchell et al., 2008). This evidence supports recent proposals that learning and retention are performed by different networks of the brain Thus, such memory processing may not require the regulation of cortical synchrony provided by the transthalamic pathways via the MDmc (at the least). Instead the direct cortico-cortical connections coursing within the PFC and across the medial temporal lobes are sufficient to support retention memory.

Widespread global amnesia associated with anterograde and retrograde memory deficits may be caused by widespread damage to subcortical structures. For example, the combined bilateral lesion damage to MDmc and fornix results in both retrograde and anterograde amnesia of OIP discriminations (Mitchell et al., 2008). This combined damage would have very likely resulted in extensive damage to interconnected regions of the medial diencephalon, medial temporal lobes, cingulate cortex and the PFC. In other primate animal models, similar types of global amnesia are also reported after combined lesions causing disconnection to the temporal stem, amygdala, and fornix (Gaffan et al., 2001; Easton et al., 2002; Gaffan, 2005). These lesions combining gray matter and white matter tracts disrupt widespread cortical—subcortical interconnections from basal forebrain, medial thalamus, and the midbrain, as well as cortico-cortical communication linking temporal and prefrontal cortices. Similar types of global amnesia are reported following widespread damage in the brain [e.g., in Korsakoff's syndrome patients (Kopelman et al., 1999; Harding et al., 2000)].

### Future directions

Further and combined behavioral, cognitive, and electrophysiology studies are required to gain greater understanding of the impact of disconnection lesions to the PFC, MD, and other interconnected structures. This research may also have clinical application in understanding the roles of the different subdivisions of the MD in many neuropsychological disorders (e.g., schizophrenia, obsessive compulsive disorder, and major depression). For example, recent studies across different species (Leal-Campanario et al., 2007, 2013; Cross et al., 2012; Parnaudeau et al., 2013) have highlighted the importance of MD-PFC communication within these interconnected neural circuits for successful cognition. Furthermore, many other studies have shown how different types of damage to brain structures interconnected to the MD can produce surprising results across species. Schoenbaum and colleagues (Stalnaker et al., 2007) have shown in rodents how the orbital frontal cortex (OFC) and amygdala interact in reversal learning tasks, with amygdala lesions abolishing the OFC dependent reversal impairments. Interestingly though, in macaques, amygdala lesions do not impair reversal learning (Izquierdo and Murray, 2007), nor do excitotoxic lesions to the OFC, however, transection of the white matter tract fibers leading into the OFC do disrupt reversals and inhibitory control (Rudebeck et al., 2013). It remains an empirical question about the extent of reversal learning deficits linked to the MD and how the MD interacts within this neural network.

There needs to be more research on the understanding of the functional consequences of the communication links between the MD and PFC related to this higher order information transfer (Guillery and Sherman, 2002). For example, how does the MD influence the neural circuitry involved for new learning yet appear to have little impact on retention. The importance of understanding the metabotropic glutamate communication between the MD and the PFC may be particularly relevant for answering this, given that glutamate invokes synaptic plasticity and potentially learning and memory due to the prolonged response of the metabotropic glutamate receptor activation (Sherman, 2013).

Finally, advances in neuroimaging are also illustrating the interconnections of the subcortical brain structures *in vivo*. For example, the fiber pathways from ventral PFC to MD have recently been documented using magnetic resonance scanning (Lehman et al., 2011). Recent DTI studies have started revealing structural connectivity of MD to PFC and limbic cortical areas and the subcortical caudate nucleus suggestive of the existence of basal ganglia-thalamo-cortical circuits in humans *in vivo* (Draganski et al., 2008; Metzger et al., 2010; Eckert et al., 2012). These advances in neuroimaging and future research that combines different behavioral and cognitive neuroscience techniques in humans and in animal models will further advance our understanding of the key roles that the subdivisions of the MD contribute to cognition.

### Conflict of interest statement

The authors declare that the research was conducted in the absence of any commercial or financial relationships that could be construed as a potential conflict of interest.

## Footnotes

^1^In the direct pathway, the neurotransmitter GABA is activated, which inhibits pallidal and nigral neurons and consequently disinhibits the thalamus and midbrain targets. In the traditional model, it is proposed that this pathway facilitates thalamocortical activity and behavioral and motor outputs. The cells express mainly dopamine D1 receptors in the direct pathway. In the indirect pathway, the neurotransmitters GABA and glutamate are activated. The GABA inhibits the pallidal neurons, which leads to less inhibition at the subthalamic level where glutamate has stronger activity levels as a result. The glutamate then influences GABA in the output neurons of the internal segment of globus pallidus and pars reticulata of the SN, which results in stronger inhibition of the thalamic and midbrain targets. It is proposed this indirect pathway then exerts an inhibitory influence on the thalamus and midbrain, equating to suppression of behavioral and motor outputs. The cells express mainly dopamine D2 receptors in the indirect pathway. These two hypotheses on the direct and indirect pathway modulation are particularly relevant to theories of motor output problems associated with Parkinson's and Huntingdon's diseases. Tekin and Cummings (2002).

## References

[B1] Aggleton J. P. Brown M. W. (1999). Episodic memory, amnesia, and the hippocampal-anterior thalamic axis. Behav. Brain Sci. 22, 425–444 discussion: 444–489. 10.1017/S0140525X99002034 11301518

[B2] Aggleton J. P. Desimone R. Mishkin M. (1986). The origin, course, and termination of the hippocampothalamic projections in the macaque. J. Comp. Neurol. 243, 409–421 10.1002/cne.902430310 3512627

[B3] Aggleton J. P. Dumont J. R. Warburton E. C. (2011). Unraveling the contributions of the diencephalon to recognition memory: a review. Learn. Mem. 18, 384–400 10.1101/lm.1884611 21597044 PMC3101772

[B4] Aggleton J. P. McMackin D. Carpenter K. Hornak J. Kapur N. Halpin S. (2000). Differential cognitive effects of colloid cysts in the third ventricle that spare or compromise the fornix. Brain 123(Pt 4), 800–815 10734011 10.1093/brain/123.4.800

[B5] Aggleton J. P. Mishkin M. (1983a). Memory impairments following restricted medial thalamic lesions in monkeys. Exp. Brain Res. 52, 199–209 6416884 10.1007/BF00236628

[B6] Aggleton J. P. Mishkin M. (1983b). Visual recognition impairment following medial thalamic lesions in monkeys. Neuropsychologia 21, 189–197 6877575 10.1016/0028-3932(83)90037-4

[B7] Aggleton J. P. Mishkin M. (1984). Projections of the amygdala to the thalamus in the cynomolgus monkey. J. Comp. Neurol. 222, 56–68 10.1002/cne.902220106 6321564

[B8] Aggleton J. P. Pearce J. M. (2001). Neural systems underlying episodic memory: insights from animal research. Philos. Trans. R. Soc. Lond. B Biol. Sci. 356, 1467–1482 10.1098/rstb.2001.0946 11571037 PMC1088529

[B9] Aggleton J. P. Shaw C. (1996). Amnesia and recognition memory: a re-analysis of psychometric data. Neuropsychologia 34, 51–62 10.1016/0028-393200150-6 8852693

[B12a] Alexander G. E. Fuster J. M. (1973). Effects of cooling prefrontal cortex on cell firing in the nucleus medialis dorsalis. Brain Res. 61, 93–105 10.1016/0006-8993(73)90518-0 4204131

[B10] Alexander G. E. DeLong M. R. Strick P. L. (1986). Parallel organization of functionally segregated circuits linking basal ganglia and cortex. Annu. Rev. Neurosci. 9, 357–381 10.1146/annurev.ne.09.030186.002041 3085570

[B11] Alexinsky T. (2001). Differential effect of thalamic and cortical lesions on memory systems in the rat. Behav. Brain Res. 122, 175–191 10.1016/S0166-432800182-6 11334648

[B12] Bachevalier J. Meunier M. Lu M. X. Ungerleider L. G. (1997). Thalamic and temporal cortex input to medial prefrontal cortex in rhesus monkeys. Exp. Brain Res. 115, 430–444 10.1007/PL00005713 9262198

[B13] Barbas H. Henion T. H. Dermon C. R. (1991). Diverse thalamic projections to the prefrontal cortex in the rhesus monkey. J. Comp. Neurol. 313, 65–94 10.1002/cne.903130106 1761756

[B14] Baxter M. G. Browning P. G. Mitchell A. S. (2008). Perseverative interference with object-in-place scene learning in rhesus monkeys with bilateral ablation of ventrolateral prefrontal cortex. Learn. Mem. 15, 126–132 10.1101/lm.804508 18299439 PMC2275654

[B15] Baxter M. G. Gaffan D. Kyriazis D. A. Mitchell A. S. (2009). Ventrolateral prefrontal cortex is required for performance of a strategy implementation task but not reinforcer devaluation effects in rhesus monkeys. Eur. J. Neurosci. 29, 2049–2059 10.1111/j.1460-9568.2009.06740.x 19453635 PMC2688497

[B16] Baxter M. G. Parker A. Lindner C. C. C. Izquierdo A. D. Murray E. A. (2000). Control of response selection by reinforcer value requires interaction of amygdala and orbital prefrontal cortex. J. Neurosci. 20, 4311–4319 10818166 10.1523/JNEUROSCI.20-11-04311.2000 PMC6772657

[B17] Bentivoglio M. Kultas-Ilinsky K. Ilinsky I. (1993). “Limbic thalamus: structure, intrinsic organization, and connections,” in Neurobiology of Cingulate Cortex and Limbic Thalamus: a Comprehensive Handbook, eds Vogt B. A. Gabriel M. (Boston, MA: Birkhauser), 71–122

[B18] Beracochea D. J. Jaffard R. Jarrard L. E. (1989). Effects of anterior or dorsomedial thalamic ibotenic lesions on learning and memory in rats. Behav. Neural Biol. 51, 364–376 10.1016/S0163-104791000-5 2730499

[B19] Berendse H. W. Groenewegen H. J. (1990). Organization of the thalamostriatal projections in the rat, with special emphasis on the ventral striatum. J. Comp. Neurol. 299, 187–228 10.1002/cne.902990206 2172326

[B20] Block A. E. Dhanji H. Thompson-Tardif S. F. Floresco S. B. (2007). Thalamic-prefrontal cortical-ventral striatal circuitry mediates dissociable components of strategy set shifting. Cereb. Cortex 17, 1625–1636 10.1093/cercor/bhl073 16963518

[B21] Brown M. W. Xiang J. Z. (1998). Recognition memory: neuronal substrates of the judgement of prior occurrence. Prog. Neurobiol. 55, 149–189 10.1016/S0301-008200002-1 9618747

[B22] Browning P. G. Easton A. Gaffan D. (2007). Frontal-temporal disconnection abolishes object discrimination learning set in macaque monkeys. Cereb. Cortex 17, 859–864 10.1093/cercor/bhk039 16707734

[B23] Burk J. A. Mair R. G. (1998). Thalamic amnesia reconsidered: excitotoxic lesions of the intralaminar nuclei, but not the mediodorsal nucleus, disrupt place delayed matching-to-sample performance in rats (*Rattus norvegicus*). Behav. Neurosci. 112, 54–67 10.1037/0735-7044.112.1.54 9517815

[B24] Burwell R. D. Witter M. P. Amaral D. G. (1995). Perirhinal and postrhinal cortices of the rat: a review of the neuroanatomical literature and comparison with findings from the monkey brain. Hippocampus 5, 390–408 10.1002/hipo.450050503 8773253

[B25] Carlesimo G. A. Lombardi M. G. Caltagirone C. (2011). Vascular thalamic amnesia: a reappraisal. Neuropsychologia 49, 777–789 10.1016/j.neuropsychologia.2011.01.026 21255590

[B26] Chauveau F. Celerier A. Ognard R. Pierard C. Beracochea D. (2005). Effects of ibotenic acid lesions of the mediodorsal thalamus on memory: relationship with emotional processes in mice. Behav. Brain Res. 156, 215–223 10.1016/j.bbr.2004.05.026 15582107

[B27] Chauveau F. Pierard C. Corio M. Celerier A. Christophe T. Vouimba R. M. (2009). Mediodorsal thalamic lesions block the stress-induced inversion of serial memory retrieval pattern in mice. Behav. Brain Res. 203, 270–278 10.1016/j.bbr.2009.05.014 19464320

[B28] Chudasama Y. (2011). Animal models of prefrontal-executive function. Behav. Neurosci. 125, 327–343 10.1037/a0023766 21639603

[B29] Chudasama Y. Bussey T. J. Muir J. L. (2001). Effects of selective thalamic and prelimbic cortex lesions on two types of visual discrimination and reversal learning. Eur. J. Neurosci. 14, 1009–1020 10.1046/j.0953-816x.2001.01607.x 11595039

[B30] Chudasama Y. Muir J. L. (2001). Visual attention in the rat: a role for the prelimbic cortex and thalamic nuclei. Behav. Neurosci. 115, 417–428 11345966

[B31] Cipolotti L. Husain M. Crinion J. Bird C. M. Khan S. S. Losseff N. (2008). The role of the thalamus in amnesia: a tractography, high-resolution MRI and neuropsychological study. Neuropsychologia 46, 2745–2758 10.1016/j.neuropsychologia.2008.05.009 18597798

[B32] Corbit L. H. Muir J. L. Balleine B. W. (2003). Lesions of mediodorsal thalamus and anterior thalamic nuclei produce dissociable effects on instrumental conditioning in rats. Eur. J. Neurosci. 18, 1286–1294 10.1046/j.1460-9568.2003.02833.x 12956727

[B33] Cross L. Brown M. W. Aggleton J. P. Warburton E. C. (2012). The medial dorsal thalamic nucleus and the medial prefrontal cortex of the rat function together to support associative recognition and recency but not item recognition. Learn. Mem. 20, 41–50 10.1101/lm.028266.112 23263843 PMC3533127

[B34] Dean P. Weiskrantz L. (1974). Loss of preoperative habits in Rhesus monkeys with inferotemporal lesions: recognition failure or relearning deficit. Neuropsychologia 12, 299–311 4472470 10.1016/0028-3932(74)90045-1

[B35] Dolleman-van der Weel M. J. Morris R. G. Witter M. P. (2009). Neurotoxic lesions of the thalamic reuniens or mediodorsal nucleus in rats affect non-mnemonic aspects of watermaze learning. Brain Struct. Funct. 213, 329–342 10.1007/s00429-008-0200-6 19132385

[B36] Draganski B. Kherif F. Kloppel S. Cook P. A. Alexander D. C. Parker G. J. (2008). Evidence for segregated and integrative connectivity patterns in the human basal ganglia. J. Neurosci. 28, 7143–7152 10.1523/JNEUROSCI.1486-08.2008 18614684 PMC6670486

[B37] Dudchenko P. A. (2001). How do animals actually solve the T maze. Behav. Neurosci. 115, 850–860 11508724

[B38] Easton A. Ridley R. M. Baker H. F. Gaffan D. (2002). Unilateral lesions of the cholinergic basal forebrain and fornix in one hemisphere and inferior temporal cortex in the opposite hemisphere produce severe learning impairments in rhesus monkeys. Cereb. Cortex 12, 729–736 10.1093/cercor/12.7.729 12050084

[B39] Eckert U. Metzger C. D. Buchmann J. E. Kaufmann J. Osoba A. Li M. (2012). Preferential networks of the mediodorsal nucleus and centromedian-parafascicular complex of the thalamus–a DTI tractography study. Hum. Brain Mapp. 33, 2627–2637 10.1002/hbm.21389 21932264 PMC6870490

[B40] Edelstyn N. M. Ellis S. J. Jenkinson P. Sawyer A. (2002). Contribution of the left dorsomedial thalamus to recognition memory: a neuropsychological case study. Neurocase 8, 442–452 10.1076/neur.8.5.442.16180 12529453

[B41] Eleore L. Lopez-Ramos J. C. Guerra-Narbona R. Delgado-Garcia J. M. (2011). Role of reuniens nucleus projections to the medial prefrontal cortex and to the hippocampal pyramidal CA1 area in associative learning. PLoS ONE 6:e23538 10.1371/journal.pone.0023538 21858159 PMC3156136

[B42] Erickson S. L. Lewis D. A. (2004). Cortical connections of the lateral mediodorsal thalamus in cynomolgus monkeys. J. Comp. Neurol. 473, 107–127 10.1002/cne.20084 15067722

[B43] Erickson S. L. Melchitzky D. S. Lewis D. A. (2004). Subcortical afferents to the lateral mediodorsal thalamus in cynomolgus monkeys. Neuroscience 129, 675–690 10.1016/j.neuroscience.2004.08.016 15541889

[B44] Fahy F. L. Riches I. P. Brown M. W. (1993). Neuronal signals of importance to the performance of visual recognition memory tasks: evidence from recordings of single neurones in the medial thalamus of primates. Prog. Brain Res. 95, 401–416 10.1016/S0079-612360384-2 8493348

[B45] Floresco S. B. Braaksma D. N. Phillips A. G. (1999). Thalamic-cortical-striatal circuitry subserves working memory during delayed responding on a radial arm maze. J. Neurosci. 19, 11061–11071 10594086 10.1523/JNEUROSCI.19-24-11061.1999 PMC6784943

[B46] Funahashi S. Takeda K. Watanabe Y. (2004). Neural mechanisms of spatial working memory: contributions of the dorsolateral prefrontal cortex and the thalamic mediodorsal nucleus. Cogn. Affect. Behav. Neurosci. 4, 409–420 10.3758/CABN.4.4.409 15849887

[B47] Fuster J. M. (2008). The Prefrontal Cortex, 4th Edn. London: Academic Press

[B48] Gaffan D. (2005). Neuroscience. Widespread cortical networks underlie memory and attention. Science 309, 2172–2173 10.1126/science.1119445 16195447

[B49] Gaffan D. Easton A. Parker A. (2002). Interaction of inferior temporal cortex with frontal cortex and basal forebrain: double dissociation in strategy implementation and associative learning. J. Neurosci. 22, 7288–7296 12177224 10.1523/JNEUROSCI.22-16-07288.2002 PMC6757896

[B50] Gaffan D. Murray E. A. (1990). Amygdalar interaction with the mediodorsal nucleus of the thalamus and the ventromedial prefrontal cortex in stimulus reward associative learning in the monkey. J. Neurosci. 10, 3479–3493 2230939 10.1523/JNEUROSCI.10-11-03479.1990 PMC6570092

[B51] Gaffan D. Murray E. A. Fabrethorpe M. (1993). Interaction of the amygdala with the frontal-lobe in reward memory. Eur. J. Neurosci. 5, 968–975 10.1111/j.1460-9568.1993.tb00948.x 8281307

[B52] Gaffan D. Parker A. (2000). Mediodorsal thalamic function in scene memory in rhesus monkeys. Brain 123, 816–827 10.1093/brain/123.4.816 10734012

[B53] Gaffan D. Parker A. Easton A. (2001). Dense amnesia in the monkey after transection of fornix, amygdala and anterior temporal stem. Neuropsychologia 39, 51–70 10.1016/S0028-393200097-X 11115655

[B54] Gaffan D. Watkins S. (1991). Mediodorsal thalamic lesions impair long-term visual associative memory in macaques. Eur. J. Neurosci. 3, 615–620 10.1111/j.1460-9568.1991.tb00847.x 12106469

[B55] Gibb S. J. Wolff M. Dalrymple-Alford J. C. (2006). Odour-place paired-associate learning and limbic thalamus: comparison of anterior, lateral and medial thalamic lesions. Behav. Brain Res. 172, 155–168 10.1016/j.bbr.2006.05.017 16769133

[B56] Giguere M. Goldman-Rakic P. S. (1988). Mediodorsal nucleus: areal, laminar, and tangential distribution of afferents and efferents in the frontal lobe of rhesus monkeys. J. Comp. Neurol. 277, 195–213 10.1002/cne.902770204 2466057

[B57] Gimenez-Amaya J. M. McFarland N. R. de las Heras S. Haber S. N. (1995). Organization of thalamic projections to the ventral striatum in the primate. J. Comp. Neurol. 354, 127–149 10.1002/cne.903540109 7542290

[B58] Goldman-Rakic P. S. Porrino L. J. (1985). The primate mediodorsal (MD) nucleus and its projection to the frontal lobe. J. Comp. Neurol. 242, 535–560 10.1002/cne.902420406 2418080

[B59] Groenewegen H. J. (1988). Organization of the afferent connections of the mediodorsal thalamic nucleus in the rat, related to the mediodorsal-prefrontal topography. Neuroscience 24, 379–431 10.1016/0306-452290339-9 2452377

[B60] Groenewegen H. J. Berendse H. W. Haber S. N. (1993). Organization of the output of the ventral striatopallidal system in the rat: ventral pallidal efferents. Neuroscience 57, 113–142 10.1016/0306-452290115-V 8278047

[B61] Groenewegen H. J. Berendse H. W. Wolters J. G. Lohman A. H. (1990). The anatomical relationship of the prefrontal cortex with the striatopallidal system, the thalamus and the amygdala: evidence for a parallel organization. Prog. Brain Res. 85, 95–116 discussion: 116–118. 2094917 10.1016/s0079-6123(08)62677-1

[B62] Groenewegen H. J. Galis-de Graaf Y. Smeets W. J. (1999a). Integration and segregation of limbic cortico-striatal loops at the thalamic level: an experimental tracing study in rats. J. Chem. Neuroanat. 16, 167–185 10422737 10.1016/s0891-0618(99)00009-5

[B63] Groenewegen H. J. Wright C. I. Beijer A. V. Voorn P. (1999b). Convergence and segregation of ventral striatal inputs and outputs. Ann. N.Y. Acad. Sci. 877, 49–63 10415642 10.1111/j.1749-6632.1999.tb09260.x

[B64] Groenewegen H. J. Wright C. I. Uylings H. B. (1997). The anatomical relationships of the prefrontal cortex with limbic structures and the basal ganglia. J. Psychopharmacol. 11, 99–106 10.1177/026988119701100202 9208373

[B65] Guillery R. W. (1995). Anatomical evidence concerning the role of the thalamus in corticocortical communication: a brief review. J. Anat. 187(Pt 3), 583–592 8586557 PMC1167461

[B66] Guillery R. W. Sherman S. M. (2002). Thalamic relay functions and their role in corticocortical communication: generalizations from the visual system. Neuron 33, 163–175 10.1016/S0896-627300582-7 11804565

[B67] Haber S. McFarland N. R. (2001). The place of the thalamus in frontal cortical-basal ganglia circuits. Neuroscientist 7, 315–324 10.1177/107385840100700408 11488397

[B68] Haber S. N. Calzavara R. (2009). The cortico-basal ganglia integrative network: the role of the thalamus. Brain Res. Bull. 78, 69–74 10.1016/j.brainresbull.2008.09.013 18950692 PMC4459637

[B69] Haber S. N. Groenewegen H. J. Grove E. A. Nauta W. J. (1985). Efferent connections of the ventral pallidum: evidence of a dual striato pallidofugal pathway. J. Comp. Neurol. 235, 322–335 10.1002/cne.902350304 3998213

[B70] Haber S. N. Knutson B. (2010). The reward circuit: linking primate anatomy and human imaging. Neuropsychopharmacology 35, 4–26 10.1038/npp.2009.129 19812543 PMC3055449

[B71] Hallanger A. E. Levey A. I. Lee H. J. Rye D. B. Wainer B. H. (1987). The origins of cholinergic and other subcortical afferents to the thalamus in the rat. J. Comp. Neurol. 262, 105–124 10.1002/cne.902620109 2442206

[B72] Hannesson D. K. Vacca G. Howland J. G. Phillips A. G. (2004). Medial prefrontal cortex is involved in spatial temporal order memory but not spatial recognition memory in tests relying on spontaneous exploration in rats. Behav. Brain Res. 153, 273–285 10.1016/j.bbr.2003.12.004 15219729

[B73] Harding A. Halliday G. Caine D. Kril J. (2000). Degeneration of anterior thalamic nuclei differentiates alcoholics with amnesia. Brain 123, 141–154 10.1093/brain/123.1.141 10611128

[B74] Harrison L. M. Mair R. G. (1996). A comparison of the effects of frontal cortical and thalamic lesions on measures of spatial learning and memory in the rat. Behav. Brain Res. 75, 195–206 10.1016/0166-432800173-8 8800656

[B75] Hunt P. R. Aggleton J. P. (1991). Medial dorsal thalamic lesions and working memory in the rat. Behav. Neural Biol. 55, 227–246 10.1016/0163-104780141-Z 2059189

[B76] Hunt P. R. Aggleton J. P. (1998a). An examination of the spatial working memory deficit following neurotoxic medial dorsal thalamic lesions in rats. Behav. Brain Res. 97, 129–141 9867238 10.1016/s0166-4328(98)00033-3

[B77] Hunt P. R. Aggleton J. P. (1998b). Neurotoxic lesions of the dorsomedial thalamus impair the acquisition but not the performance of delayed matching to place by rats: a deficit in shifting response rules. J. Neurosci. 18, 10045–10052 9822759 10.1523/JNEUROSCI.18-23-10045.1998 PMC6793303

[B78] Hunt P. R. Neave N. Shaw C. Aggleton J. P. (1994). The effects of lesions to the fornix and dorsomedial thalamus on concurrent discrimination learning by rats. Behav. Brain Res. 62, 195–205 10.1016/0166-432890028-0 7945970

[B79] Isseroff A. Rosvold H. E. Galkin T. W. Goldman-Rakic P. S. (1982). Spatial memory impairments following damage to the mediodorsal nucleus of the thalamus in rhesus monkeys. Brain Res. 232, 97–113 10.1016/0006-899390613-8 7034865

[B80] Izquierdo A. Murray E. A. (2007). Selective bilateral amygdala lesions in rhesus monkeys fail to disrupt object reversal learning. J. Neurosci. 27, 1054–1062 10.1523/JNEUROSCI.3616-06.2007 17267559 PMC6673199

[B81] Izquierdo A. Murray E. A. (2010). Functional interaction of medial mediodorsal thalamic nucleus but not nucleus accumbens with amygdala and orbital prefrontal cortex is essential for adaptive response selection after reinforcer devaluation. J. Neurosci. 30, 661–669 10.1523/JNEUROSCI.3795-09.2010 20071531 PMC2835504

[B82] Jones E. G. (1985). The Thalamus. New York, NY: Plenum

[B83] Jones E. G. (ed.). (1998). The Thalamus of Primates. Amsterdam: Elsevier

[B84] Kopelman M. D. (1995). The Korsakoff syndrome. Br. J. Psychiatry 166, 154–173 10.1192/bjp.166.2.154 7728359

[B85] Kopelman M. D. (2002). Disorders of memory. Brain 125, 2152–2190 10.1093/brain/awf229 12244076

[B86] Kopelman M. D. Stanhope N. Kingsley D. (1997). Temporal and spatial context memory in patients with focal frontal, temporal lobe, and diencephalic lesions. Neuropsychologia 35, 1533–1545 10.1016/S0028-393200076-6 9460723

[B87] Kopelman M. D. Stanhope N. Kingsley D. (1999). Retrograde amnesia in patients with diencephalic, temporal lobe or frontal lesions. Neuropsychologia 37, 939–958 10.1016/S0028-393200143-2 10426519

[B88] Kornecook T. J. Anzarut A. Pinel J. P. (1999). Rhinal cortex, but not medial thalamic, lesions cause retrograde amnesia for objects in rats. Neuroreport 10, 2853–2858 10.1097/00001756-199909090-00028 10511452

[B89] Krazem A. Beracochea D. Jaffard R. (1995). Effects of mammillary bodies and mediodorsal thalamic lesions on the acquisition and retention of a learning set in mice: paradoxical effect of the intersession interval. Behav. Brain Res. 67, 51–58 10.1016/0166-432800103-M 7748500

[B90] Krettek J. E. Price J. L. (1977). The cortical projections of the mediodorsal nucleus and adjacent thalamic nuclei in the rat. J. Comp. Neurol. 171, 157–191 10.1002/cne.901710204 64477

[B91] Kril J. J. Halliday G. M. (1999). Brain shrinkage in alcoholics: a decade on and what have we learned. Prog. Neurobiol. 58, 381–387 10368034 10.1016/s0301-0082(98)00091-4

[B92] Leal-Campanario R. Delgado-Garcia J. M. Gruart A. (2013). The rostral medial prefrontal cortex regulates the expression of conditioned eyelid responses in behaving rabbits. J. Neurosci. 33, 4378–4386 10.1523/JNEUROSCI.5560-12.2013 23467354 PMC6704970

[B93] Leal-Campanario R. Fairen A. Delgado-Garcia J. M. Gruart A. (2007). Electrical stimulation of the rostral medial prefrontal cortex in rabbits inhibits the expression of conditioned eyelid responses but not their acquisition. Proc. Natl. Acad. Sci. U.S.A. 104, 11459–11464 10.1073/pnas.0704548104 17592148 PMC1899194

[B94] Lehman J. F. Greenberg B. D. McIntyre C. C. Rasmussen S. A. Haber S. N. (2011). Rules ventral prefrontal cortical axons use to reach their targets: implications for diffusion tensor imaging tractography and deep brain stimulation for psychiatric illness. J. Neurosci. 31, 10392–10402 10.1523/JNEUROSCI.0595-11.2011 21753016 PMC3445013

[B95] Lopez J. Wolff M. Lecourtier L. Cosquer B. Bontempi B. Dalrymple-Alford J. (2009). The intralaminar thalamic nuclei contribute to remote spatial memory. J. Neurosci. 29, 3302–3306 10.1523/JNEUROSCI.5576-08.2009 19279267 PMC6666443

[B96] Malkova L. Gaffan D. Murray E. A. (1997). Excitotoxic lesions of the amygdala fail to produce impairment in visual learning for auditory secondary reinforcement but interfere with reinforcer devaluation effects in rhesus monkeys. J. Neurosci. 17, 6011–6020 9221797 10.1523/JNEUROSCI.17-15-06011.1997 PMC6573210

[B97] McFarland N. R. Haber S. N. (2002). Thalamic relay nuclei of the basal ganglia form both reciprocal and nonreciprocal cortical connections, linking multiple frontal cortical areas. J. Neurosci. 22, 8117–8132 12223566 10.1523/JNEUROSCI.22-18-08117.2002 PMC6758100

[B98] Metzger C. D. Eckert U. Steiner J. Sartorius A. Buchmann J. E. Stadler J. (2010). High field FMRI reveals thalamocortical integration of segregated cognitive and emotional processing in mediodorsal and intralaminar thalamic nuclei. Front. Neuroanat. 4:138 10.3389/fnana.2010.00138 21088699 PMC2981419

[B99] M'Harzi M. Jarrard L. E. Willig F. Palacios A. Delacour J. (1991). Selective fimbria and thalamic lesions differentially impair forms of working memory in rats. Behav. Neural Biol. 56, 221–239 10.1016/0163-104790364-V 1759943

[B100] Mitchell A. S. Baxter M. G. Gaffan D. (2007a). Dissociable performance on scene learning and strategy implementation after lesions to magnocellular mediodorsal thalamic nucleus. J. Neurosci. 27, 11888–11895 17978029 10.1523/JNEUROSCI.1835-07.2007 PMC2241732

[B101] Mitchell A. S. Browning P. G. Baxter M. G. (2007b). Neurotoxic lesions of the medial mediodorsal nucleus of the thalamus disrupt reinforcer devaluation effects in rhesus monkeys. J. Neurosci. 27, 11289–11295 17942723 10.1523/JNEUROSCI.1914-07.2007 PMC2242856

[B102] Mitchell A. S. Browning P. G. Wilson C. R. Baxter M. G. Gaffan D. (2008). Dissociable roles for cortical and subcortical structures in memory retrieval and acquisition. J. Neurosci. 28, 8387–8396 10.1523/JNEUROSCI.1924-08.2008 18716197 PMC6671048

[B103] Mitchell A. S. Dalrymple-Alford J. C. (2005). Dissociable memory effects after medial thalamus lesions in the rat. Eur. J. Neurosci. 22, 973–985 10.1111/j.1460-9568.2005.04199.x 16115220

[B104] Mitchell A. S. Dalrymple-Alford J. C. (2006). Lateral and anterior thalamic lesions impair independent memory systems. Learn. Mem. 13, 388–396 10.1101/lm.122206 16741289 PMC1475822

[B105] Mitchell A. S. Gaffan D. (2008). The magnocellular mediodorsal thalamus is necessary for memory acquisition, but not retrieval. J. Neurosci. 28, 258–263 10.1523/JNEUROSCI.4922-07.2008 18171943 PMC6671139

[B106] Mitchell J. B. Laiacona J. (1998). The medial frontal cortex and temporal memory: tests using spontaneous exploratory behaviour in the rat. Behav. Brain Res. 97, 107–113 10.1016/S0166-432800032-1 9867236

[B107] Moreau P. H. Tsenkina Y. Lecourtier L. Lopez J. Cosquer B. Wolff M. (2013). Lesions of the anterior thalamic nuclei and intralaminar thalamic nuclei: place and visual discrimination learning in the water maze. Brain Struct. Funct. 218, 657–667 10.1007/s00429-012-0419-0 22543509

[B108] Morris R. G. (2001). Episodic-like memory in animals: psychological criteria, neural mechanisms and the value of episodic-like tasks to investigate animal models of neurodegenerative disease. Philos. Trans. R. Soc. Lond. B Biol. Sci. 356, 1453–1465 10.1098/rstb.2001.0945 11571036 PMC1088528

[B109] Mumby D. G. Pinel J. P. Dastur F. N. (1993). Mediodorsal thalamic lesions impair object recognition in rats. Psychobiology 21, 27–36

[B110] Murray E. A. Gaffan D. (2006). Prospective memory in the formation of learning sets by rhesus monkeys (*Macaca mulatta*). J. Exp. Psychol. Anim. Behav. Process. 32, 87–90 10.1037/0097-7403.32.1.87 16435968

[B111] Neave N. Sahgal A. Aggleton J. P. (1993). Lack of effect of dorsomedial thalamic lesions on automated tests of spatial memory in the rat. Behav. Brain Res. 55, 39–49 10.1016/0166-432890005-B 8329125

[B112] Negyessy L. Hamori J. Bentivoglio M. (1998). Contralateral cortical projection to the mediodorsal thalamic nucleus: origin and synaptic organization in the rat. Neuroscience 84, 741–753 10.1016/S0306-452200559-9 9579780

[B113a] Olszewski J. (1952). The Thalamus of the Macaca mulatta. An Atlas for Use with the stereotaxic Instrument. Basel: Karger

[B113] Ostlund S. B. Balleine B. W. (2008). Differential involvement of the basolateral amygdala and mediodorsal thalamus in instrumental action selection. J. Neurosci. 28, 4398–4405 10.1523/JNEUROSCI.5472-07.2008 18434518 PMC2652225

[B114a] Parker A. Gaffan D. (1998). Interaction of frontal and perirhinal cortices in visual object recognition memory in monkeys. Eur. J. Neurosci. 10, 3044–3057 10.1046/j.1460-9568.1998.00306.x 9786199

[B114] Parker A. Eacott M. J. Gaffan D. (1997). The recognition memory deficit caused by mediodorsal thalamic lesion in non-human primates: a comparison with rhinal cortex lesion. Eur. J. Neurosci. 9, 2423–2431 10.1111/j.1460-9568.1997.tb01659.x 9464936

[B115] Parnaudeau S. O'Neill P. K. Bolkan S. S. Ward R. D. Abbas A. I. Roth B. L. (2013). Inhibition of mediodorsal thalamus disrupts thalamofrontal connectivity and cognition. Neuron 77, 1151–1162 10.1016/j.neuron.2013.01.038 23522049 PMC3629822

[B116a] Paxinos G. Watson C. (1998). The Rat Brain: in Stereotaxic Coordinates. Sydney: Academic Press

[B116] Peinado-Manzano M. A. Pozo-Garcia R. (1991). The role of different nuclei of the thalamus in processing episodic information. Behav. Brain Res. 45, 17–27 10.1016/S0166-432880176-7 1764201

[B117] Peinado-Manzano M. A. Pozo-Garcia R. (1996). Retrograde amnesia in rats with dorsomedial thalamic damage. Behav. Brain Res. 80, 177–184 10.1016/0166-432800033-2 8905141

[B118] Pergola G. Gunturkun O. Koch B. Schwarz M. Daum I. Suchan B. (2012). Recall deficits in stroke patients with thalamic lesions covary with damage to the parvocellular mediodorsal nucleus of the thalamus. Neuropsychologia 50, 2477–2491 10.1016/j.neuropsychologia.2012.06.019 22750446

[B119] Pickens C. L. (2008). A limited role for mediodorsal thalamus in devaluation tasks. Behav. Neurosci. 122, 659–676 10.1037/0735-7044.122.3.659 18513136 PMC3115662

[B120] Preuss T. M. Goldman-Rakic P. S. (1987). Crossed corticothalamic and thalamocortical connections of macaque prefrontal cortex. J. Comp. Neurol. 257, 269–281 10.1002/cne.902570211 3571529

[B121] Price J. L. (ed.). (1995). Thalamus, 2nd Edn. Sydney, NSW: Academic Press, Inc.

[B122] Ray J. P. Price J. L. (1993). The organization of projections from the mediodorsal nucleus of the thalamus to orbital and medial prefrontal cortex in Macaque monkeys. J. Comp. Neurol. 337, 1–31 10.1002/cne.903370102 7506270

[B123] Ridley R. M. Baker H. F. Cummings R. M. Green M. E. Leow-Dyke A. (2005). Mild topographical memory impairment following crossed unilateral lesions of the mediodorsal thalamic nucleus and the inferotemporal cortex. Behav. Neurosci. 119, 518–525 10.1037/0735-7044.119.2.518 15839798

[B124] Ridley R. M. Maclean C. J. Young F. M. Baker H. F. (2002). Learning impairments in monkeys with combined but not separate excitotoxic lesions of the anterior and mediodorsal thalamic nuclei. Brain Res. 950, 39–51 10.1016/S0006-899302984-0 12231227

[B125] Rudebeck P. H. Saunders R. C. Prescott A. T. Chau L. S. Murray E. A. (2013). Prefrontal mechanisms of behavioral flexibility, emotion regulation and value updating. Nat. Neurosci. 16, 1140–1145 10.1038/nn.3440 23792944 PMC3733248

[B126] Russchen F. T. Amaral D. G. Price J. L. (1987). The afferent input to the magnocellular division of the mediodorsal thalamic nucleus in the monkey, *Macaca fascicularis*. J. Comp. Neurol. 256, 175–210 10.1002/cne.902560202 3549796

[B127] Saalmann Y. B. Pinsk M. A. Wang L. Li X. Kastner S. (2012). The pulvinar regulates information transmission between cortical areas based on attention demands. Science 337, 753–756 10.1126/science.1223082 22879517 PMC3714098

[B128] Sanchez-Gonzalez M. A. Garcia-Cabezas M. A. Rico B. Cavada C. (2005). The primate thalamus is a key target for brain dopamine. J. Neurosci. 25, 6076–6083 10.1523/JNEUROSCI.0968-05.2005 15987937 PMC6725054

[B129] Saunders R. C. Mishkin M. Aggleton J. P. (2005). Projections from the entorhinal cortex, perirhinal cortex, presubiculum, and parasubiculum to the medial thalamus in macaque monkeys: identifying different pathways using disconnection techniques. Exp. Brain Res. 167, 1–16 10.1007/s00221-005-2361-3 16143859

[B130] Schmahmann J. D. (2003). Vascular syndromes of the thalamus. Stroke 34, 2264–2278 10.1161/01.STR.0000087786.38997.9E 12933968

[B131] Sherman S. M. (2005). Thalamic relays and cortical functioning. Prog. Brain Res. 149, 107–126 10.1016/S0079-612349009-3 16226580

[B132] Sherman S. M. (2007). The thalamus is more than just a relay. Curr. Opin. Neurobiol. 17, 417–422 10.1016/j.conb.2007.07.003 17707635 PMC2753250

[B133] Sherman S. M. (2013). The function of metabotropic glutamate receptors in thalamus and cortex. Neuroscientist. [Epub ahead of print]. 10.1177/1073858413478490 23459618 PMC4747429

[B134a] Sherman S. M. Guillery R. W. (2002). The role of the thalamus in the flow of information to the cortex. Philos Trans R. Soc. Lond. B. Biol. Sci. 357, 1685–1708 10.1098/rstb.2002.1161 12626004 PMC1693087

[B134] Sherman S. M. Guillery R. W. (2005). Exploring the Thalamus and its Role in Cortical Function. Cambridge: The MIT Press

[B135] Sherman S. M. Guillery R. W. (2011). Distinct functions for direct and transthalamic corticocortical connections. J. Neurophysiol. 106, 1068–1077 10.1152/jn.00429.2011 21676936

[B136] Shuren J. E. Jacobs D. H. Heilman K. M. (1997). Diencephalic temporal order amnesia. J. Neurol. Neurosurg. Psychiatry 62, 163–168 10.1136/jnnp.62.2.163 9048717 PMC486728

[B137] Sommer M. A. Wurtz R. H. (2006). Influence of the thalamus on spatial visual processing in frontal cortex. Nature 444, 374–377 10.1038/nature05279 17093408

[B138] Stalnaker T. A. Franz T. M. Singh T. Schoenbaum G. (2007). Basolateral amygdala lesions abolish orbitofrontal-dependent reversal impairments. Neuron 54, 51–58 10.1016/j.neuron.2007.02.014 17408577

[B139] Stokes K. A. Best P. J. (1988). Mediodorsal thalamic lesions impair radial maze performance in the rat. Behav. Neurosci. 102, 294–300 10.1037/0735-7044.102.2.294 3365324

[B140] Stokes K. A. Best P. J. (1990a). Mediodorsal thalamic lesions impair “reference” and “working” memory in rats. Physiol. Behav. 47, 471–476 2359755 10.1016/0031-9384(90)90111-g

[B141] Stokes K. A. Best P. J. (1990b). Response biases do not underlie the radial maze deficit in rats with mediodorsal thalamus lesions. Behav. Neural Biol. 53, 334–345 2350320 10.1016/0163-1047(90)90198-f

[B142] Tanibuchi I. Goldman-Rakic P. S. (2005). Comparison of oculomotor neuronal activity in paralaminar and mediodorsal thalamus in the rhesus monkey. J. Neurophysiol. 93, 614–619 10.1152/jn.00969.2003 15306630

[B143] Tekin S. Cummings J. L. (2002). Frontal-subcortical neuronal circuits and clinical neuropsychiatry: an update. J. Psychosom. Res. 53, 647–654 10.1016/S0022-399900428-2 12169339

[B144] Thornton J. A. Rothblat L. A. Murray E. A. (1997). Rhinal cortex removal produces amnesia for preoperatively learned discrimination problems but fails to disrupt postoperative acquisition and retention in rhesus monkeys. J. Neurosci. 17, 8536–8549 9334426 10.1523/JNEUROSCI.17-21-08536.1997 PMC6573729

[B145] Uylings H. B. Groenewegen H. J. Kolb B. (2003). Do rats have a prefrontal cortex. Behav. Brain Res. 146, 3–17 14643455 10.1016/j.bbr.2003.09.028

[B146] Van der Werf Y. D. Jolles J. Witter M. P. Uylings H. B. (2003a). Contributions of thalamic nuclei to declarative memory functioning. Cortex 39, 1047–1062 14584566 10.1016/s0010-9452(08)70877-3

[B147] Van der Werf Y. D. Scheltens P. Lindeboom J. Witter M. P. Uylings H. B. Jolles J. (2003b). Deficits of memory, executive functioning and attention following infarction in the thalamus; a study of 22 cases with localised lesions. Neuropsychologia 41, 1330–1344 12757906 10.1016/s0028-3932(03)00059-9

[B148] Van der Werf Y. D. Witter M. P. Uylings H. B. Jolles J. (2000). Neuropsychology of infarctions in the thalamus: a review. Neuropsychologia 38, 613–627 10.1016/S0028-393200104-9 10689038

[B149] Vann S. D. (2013). Dismantling the Papez circuit for memory in rats. Elife 2:e00736 10.7554/eLife.00736 23805381 PMC3691571

[B150] Victor M. Adams R. D. Collins G. H. (1971). The Wernicke-Korsakoff syndrome. A clinical and pathological study of 245 patients, 82 with post-mortem examinations. Contemp. Neurol. Ser. 7, 1–206 5162155

[B151] Watanabe Y. Funahashi S. (2012). Thalamic mediodorsal nucleus and working memory. Neurosci. Biobehav. Rev. 36, 134–142 10.1016/j.neubiorev.2011.05.003 21605592

[B152] Winocur G. (1985). The hippocampus and thalamus: their roles in short- and long-term memory and the effects of interference. Behav. Brain Res. 16, 135–152 10.1016/0166-432890088-9 4041213

[B153] Winocur G. (1990). Anterograde and retrograde amnesia in rats with dorsal hippocampal or dorsomedial thalamic lesions. Behav. Brain Res. 38, 145–154 10.1016/0166-432890012-4 2363834

[B154] Wolff M. Gibb S. J. Cassel J. C. Dalrymple-Alford J. C. (2008). Anterior but not intralaminar thalamic nuclei support allocentric spatial memory. Neurobiol. Learn. Mem. 90, 71–80 10.1016/j.nlm.2008.01.007 18296080

[B155] Wolff M. Gibb S. J. Dalrymple-Alford J. C. (2006). Beyond spatial memory: the anterior thalamus and memory for the temporal order of a sequence of odor cues. J. Neurosci. 26, 2907–2913 10.1523/JNEUROSCI.5481-05.2006 16540567 PMC6673972

[B156] Xiang J. Z. Brown M. W. (2004). Neuronal responses related to long-term recognition memory processes in prefrontal cortex. Neuron 42, 817–829 10.1016/j.neuron.2004.05.013 15182720

[B157] Xiao D. Zikopoulos B. Barbas H. (2009). Laminar and modular organization of prefrontal projections to multiple thalamic nuclei. Neuroscience 161, 1067–1081 10.1016/j.neuroscience.2009.04.034 19376204 PMC2700123

[B158] Yeterian E. H. Pandya D. N. (1994). Laminar origin of striatal and thalamic projections of the prefrontal cortex in rhesus monkeys. Exp. Brain Res. 99, 383–398 10.1007/BF00228975 7957718

[B159] Young H. L. Stevens A. A. Converse E. Mair R. G. (1996). A comparison of temporal decay in place memory tasks in rats (*Rattus norvegicus*) with lesions affecting thalamus, frontal cortex, or the hippocampal system. Behav. Neurosci. 110, 1244–1260 10.1037/0735-7044.110.6.1244 8986329

[B160] Zhang Y. Burk J. A. Glode B. M. Mair R. G. (1998). Effects of thalamic and olfactory cortical lesions on continuous olfactory delayed nonmatching-to-sample and olfactory discrimination in rats (*Rattus norvegicus*). Behav. Neurosci. 112, 39–53 10.1037/0735-7044.112.1.39 9517814

[B161] Zola-Morgan S. Squire L. R. (1985). Amnesia in monkeys after lesions of the mediodorsal nucleus of the thalamus. Ann. Neurol. 17, 558–564 10.1002/ana.410170605 4040731

